# Global invasion history of the emerging plant pathogen *Phytophthora multivora*

**DOI:** 10.1186/s12864-022-08363-5

**Published:** 2022-02-22

**Authors:** Tetyana Tsykun, Simone Prospero, Corine N. Schoebel, Alexander Rea, Treena I. Burgess

**Affiliations:** 1 Diversity and Evolution, Department Ecology and Evolution, Goethe-University Frankfurt am Main, Institute of Ecology, Max-von-Laue Str. 13, DE-60438 Frankfurt am Main, Germany; 2 Senckenberg Biodiversity and Climate Research Centre SBiK-F, Georg-Voigt Str. 14-16, DE-60325 Frankfurt am Main, Germany; 3 Swiss Federal Research Institute WSL, Zürcherstrasse 111, CH-8903 Birmensdorf, Switzerland; 4 Department of Diagnostic Genomics, PathWest Laboratory Medicine Western Australia, Nedlands, Western Australia Australia; 5 Phytophthora Science and Management, Harry Butler Institute, Murdoch, Perth, Australia; 6 Forestry and Agriculture Biotechnology Institute, University of Pretoria, Pretoria, 0002 South Africa

**Keywords:** Biological invasion, Center of origin, Population diversity, Bridgehead effect, Demographic history

## Abstract

**Background:**

global trade in living plants and plant material has significantly increased the geographic distribution of many plant pathogens. As a consequence, several pathogens have been first found and described in their introduced range where they may cause severe damage on naïve host species. Knowing the center of origin and the pathways of spread of a pathogen is of importance for several reasons, including identifying natural enemies and reducing further spread. Several *Phytophthora* species are well-known invasive pathogens of natural ecosystems, including *Phytophthora multivora.* Following the description of *P. multivora* from dying native vegetation in Australia in 2009, the species was subsequently found to be common in South Africa where it does not cause any remarkable disease. There are now reports of *P. multivora* from many other countries worldwide, but not as a commonly encountered species in natural environments.

**Results:**

a global collection of 335 isolates from North America, Europe, Africa, Australia, the Canary Islands, and New Zealand was used to unravel the worldwide invasion history of *P. multivora,* using 10 microsatellite markers for all isolates and sequence data from five loci from 94 representative isolates. Our population genetic analysis revealed an extremely low heterozygosity, significant non-random association of loci and substantial genotypic diversity suggesting the spread of *P. multivora* readily by both asexual and sexual propagules. The *P. multivora* populations in South Africa, Australia, and New Zealand show the most complex genetic structure, are well established and evolutionary older than those in Europe, North America and the Canary Islands.

**Conclusions:**

according to the conducted analyses, the world invasion of *P. multivora* most likely commenced from South Africa, which can be considered the center of origin of the species. The pathogen was then introduced to Australia, which acted as bridgehead population for Europe and North America. Our study highlights a complex global invasion pattern of *P. multivora*, including both direct introductions from the native population and secondary spread/introductions from bridgehead populations.

**Supplementary Information:**

The online version contains supplementary material available at 10.1186/s12864-022-08363-5.

## Background

Global trade in plants and plant products has inadvertently spread numerous plant pathogens worldwide, resulting in severe disease epidemics [1–3]. For many pathogens of agricultural crops, well-maintained databases exist, showing their current distribution [see Table 1 in 4]. With such information, the trade-associated risk of spreading the pathogen among countries or regions can be assessed [3, 4]. However, while information may be available for the geographic distribution of a pathogen, its center of origin may remain unknown [5]. Knowing where a pathogen has arisen and evolved is not only of academic importance but has concrete implications. For example, according to the enemy release hypothesis, the chances of finding natural enemies able to control a pathogen are higher in the native rather than in the introduced range [6]. Moreover, knowing the center of origin of a pathogen can help to understand the pathways of spread and to prevent or at least stop repeated introductions. The fewer the introductions, the lower the genetic diversity, and pathogens with low genetic diversity are less likely to overcome host resistance [7], increasing our chances to control or eradicate the pathogen. Similarly, for invasive pathogens in natural ecosystems, the lower the number of repeated introductions and the diversity of the pathogen, the higher the chance of finding a level of resistance within the naïve plant community [8].

**Table 1:** Multilocus genotype (MLG) summary statistics inferred from 10 SSR loci in six populations (South Africa, Australia, Canary Islands, Europe, New Zealand, and North America) and the global population of *Phytophthora multivora*

Population	Samples	MLG^**a**^	eMLG^**b**^	cMLG^**c**^	iMLG^**d**^ (%)
South Africa	43	34	9	5	29 (85)
Australia	198	54	7	10	44 (81)
Canary Islands	26	3	2	3	0 (n.a.)
Europe	12	7	6	5	2 (29)
New Zealand	20	18	10	0	18 (100)
North America	7	3	3	3	0 (0)
Global population	306	119	7	10	93 (78)

^a^Number of MLGs in each population

^b^Expected number of MLGs at the largest shared sample size (here 7 samples in North America) based on a rarefaction;

^c^Number of MLGs also present in other populations;

^d^Number MLGs unique to the specific population (in brackets as a percent of the occurring MLGs)

Many *Phytophthora* species (Oomycetes, Stramenopila) are devastating plant pathogens in agriculture [9] and natural ecosystems [10], including those in the *P. citricola* sensu *lato* complex [11]. The complex includes several morphologically similar but phylogenetically distinct species described in the following order: *P. citricola* sensu stricto, *P. plurivora* [11], *P. multivora* [12], *P. pini* [13], *P. capansis* [14], *P. pachypleura* [15], *P. acerina* [16]*, P. caryae* [17] and *P. emzansi* [18]. Many records initially ascribed to *P. citricola s.l.* have now been reassigned to other species within the complex, mainly *P. plurivora* and *P. multivora*, making its host range and species distribution narrower than previously thought [11, 12]*.*

*Phytophthora plurivora* is a widespread pathogen in temperate forests of the northern hemisphere, where it is frequently associated with root and stem diseases [19, 20]. The species is also reported on ornamental plants in European and North American nurseries [21, 22], and it was shown *P. plurivora* had been introduced to North America from Europe [23]. Moderate genetic diversity and lack of genetic population structure in the European population suggested an introduced origin, but due to incomplete sample collection, the centre of origin of the species could not be determined. In the northern hemisphere, *P. multivora* is reported to be rare and somewhat restricted to nurseries and urban plantations [24], suggesting a relatively recent introduction*.*

*Phytophthora multivora* was the first pathogenic *Phytophthora* species to be described from natural ecosystems in Australia. As it is widely distributed and associated with significant plant mortality, it was initially hypothesized to be native to Western Australia [12]. The species has since been reported on five continents, usually associated with diseases of woody plants. Reports from natural ecosystems [12, 25, 26], production orchards [27–30] and restoration sites [31, 32] are from Mediterranean climates, whereas reports from ornamentals and the nursery trade extend into temperate regions of Europe [19], North America [33] and Japan [34, 35]. The global distribution of *P. multivora* brings into doubt the assumption that it is native to Western Australia.

Investigations about the genetic diversity and comparative analysis of population structure combined with a coalescent approach can decipher demographic history and gene flow among geographic populations, which may shed light on the possible origin of a species [23, 36–38]. Thus, in order to unravel the worldwide invasion history of *P. multivora,* we obtained a global collection of isolates from North America, Europe, Africa, Australia, the Canary Islands, New Zealand, and examined them with two sets of genetic markers; firstly, 10 single sequences repeats (SSR) and secondly sequences of three mitochondrial and three nuclear loci. Specifically, we addressed the following questions. (1) How genetically diverse are the studied populations? (2) How does the genetic structure differ among the populations? (3) What was the most likely demographic history of the populations’ establishment? (4) What is the geographic origin of the isolate harboring the ancestral state sequences according to coalescent phylogenetic analysis? and (5) What was the most likely global invasion history of *P. multivora*?

## Results

### Loci and multilocus genotypes

All 10 screened SSR loci were formally polymorphic, i.e. minor allele frequencies were > 5% in the global population and > 1% in each geographic population, and minor alleles were observed in more than two samples per geographic population. Pairwise linkage disequilibrium and deviation from Hardy-Weinberg equilibrium were not consistent across loci and populations (Supplementary Fig. S1–2). Hence, all loci were considered for population genetic analyses. However, the polymorphism of SSR loci was generally very low, with six out of the 10 loci showing a distinct dominant allele with a frequency of more than 72% in the global population. Less than 0.04% of missing data (i.e. no allele at a specific locus) were observed among the 306 isolates screened; thus, all multilocus genotypes (MLGs) were included in the study.

Based on the number of expected MLGs (see eMLG in Table 1), New Zealand, South Africa, and Australia were the most diverse populations. Only 10 MLGs among 119 MLGs were present in more than one population worldwide, and remarkably, all those MLGs occurred in Australia. Whereas 18 MLGs recovered in New Zealand were unique to this population.

We successfully sequenced three mitochondrial regions NADHI, *cox*I and *cox*IGS. The *cox*I and *cox*IGS were trimmed and concatenated for the downstream analysis into mitochondrial gene region COI, and three nuclear loci (ASF, ENOLASE*,* and HSP90) loci for 93 *P. multivora* DNA isolates. Additionally, we cloned 24 ENOLASE gene variants from 8 isolates and 10 HSP90 gene variants from 3 isolates. Alignment of sequences, including cloned loci, revealed 106 informative nucleotide sites in 113 genotypes. Clone-censored per geographic population data set resulted in 60 unique genotype sequences used for further phylogeographic investigation. Sequence and site diversities between mitochondrial and nuclear gene regions were comparable; however, they differed substantially among populations (Table 2). The highest estimates of diversity were observed in the South African populations, followed by the Australian and New Zealand populations. On the other side, populations from the Canary Islands and North America revealed the lowest diversity values.

**Table 2:** The genetic diversity of nuclear and mitochondrial loci of *Phytophthora multivora* in the six populations (South Africa, Australia, Canary Islands, Europe, New Zealand, and North America) analyzed in this study

Population	Global	South Africa	Australia	Canary Islands	Europe	New Zealand	North America
Estimates ^**a**^							
Number of Sequences	113	31	37	7	15	15	8
Length, bp	3370 (m: 1192; n: 2176)						
Polymorphic sites (N)	10 (m: 41; n: 65)	73 (m: 27; n: 46)	41 (m: 16; n: 25)	4 (m: 2; n: 2)	29 (m: 11; n: 18)	25 (m: 12; n:13)	4 (m: 1; n: 3)
Pi^b^	0.0036 (m: 0.0038; n: 0.0034)	0.0047 (m: 0.0047; n: 0.0046)	0.0035 (m: 0.0037; n: 0.0034)	0.0003 (m: 0.0065; n: 0.0001)	0.0283 (m: 0.0032; n: 0.0027)	0.0024 (m: 0.0028; n: 0.0022)	0.0004 (m: 0.0043; n: 0.0003)
ϴ^c^	16.44 (m: 6.43; n: 10.02)	13.72 (m: 5.19; n: 8.53)	7.54 (m: 2.87; n: 4.67)	1.03 (m: 0.51; n: 0.51)	6.43 (m: 2.36; n: 4.07)	5.36 (m: 2.57; n: 2.79)	0.99 (m: 0.25; n: 0.74)

^a^For each diversity estimate, the total value and in brackets the values for mitochondrial loci sequences (m; COI and NADHI) and for nuclear loci sequences (n; ASP, ENOLASE, HSP90) are given

^b^Nucleotide diversity per site

^c^Theta per sequence [39]

#### Population diversity and structure

The diversity and genetic structure of the global *P. multivora* population were assessed using data from 10 SSR loci and 119 MLGs (clone-corrected data per population). The highest diversity estimates (i.e. number of MLGs, allelic richness, and diversity indexes) were observed in the South African and Australian populations, followed by the New Zealand population (Table 3). These three populations also harbored private alleles (3–5 per population). In contrast, besides the lack of private alleles, the Canary Islands, European, and North American populations each showed relatively low diversity estimates. However, MLGs found in Europe showed slightly higher diversity than MLGs from the Canary Islands or North America (Table 3).

**Table 3:** Summary statistics inferred from 10 SSR loci in the six populations (South Africa, Australia, Canary Islands, Europe, New Zealand, and North America) of *Phytophthora multivora* analyzed in this study

Population	South Africa	Australia	Canary Islands	Europe	New Zealand	North America
Samples (N)	43	198	26	12	20	7
MLGs^1^ (N)	34	54	3	7	18	3
Ar ± SD^2^	2.78 ± 0.68	2.64 ± 0.69	1.70 ± 0.46	2.16 ± 0.73	2.52 ± 0.83	1.60 ± 0.66
(Ar ± SD)	(4.3 ± 1.52)	(4.18 ± 1.45)	–	–	(3.70 ± 1.68)	–
Pa^3^	3	5	0	0	4	0
H^4^	3.53	3.99	1.10	1.95	2.89	1.10
Λ^5^	0.97	0.98	0.67	0.86	0.94	0.67
Hexp^6^	0.60	0.56	0.37	0.43	0.51	0.29
Hobs^7^	0.00	0.02	0.00	0.00	0.00	0.00
I_A_^8^	0.91	0.88	3.43	1.30	1.05	3.00
(*p*-value)	(0.00)	(0.00)	(0.01)	(0.00)	(0.00)	(0.00)
rD^9^	0.10	0.10	0.57	0.16	0.12	1.00
(*p*-value)	(0.00)	(0.00)	(0.02)	(0.00)	(0.00)	(0.03)

^1^Number of multilocus genotypes in each population;

^2^Mean allelic richness and standard deviation computed per locus and rarefied to the population with the lowest sample size (North America). In brackets, mean allelic richness computed for populations with more than 10 MLGs;

^3^Private alleles observed in each population;

^4^Shannon-Weiner diversity index;

^5^Simpson’s diversity index;

^6^Nei’s gene diversity (expected heterozygosity);

^7^Observed heterozygosity;

^8^Index of association for each population with *P-*value (in brackets) resulting from a one-sided permutation test;

^9^Standardized index of association for each population with *P-*value (in brackets) resulting from a one-sided permutation test

All populations showed no heterozygosity, except the population in Australia in which two MLGs were heterozygous in five loci each. The index of association (I_A_) and the standardized index of association (rD) indicated a significant (*P* < 0.05) non-random association of loci and departure from panmixia in all populations (Table 3).

Significant population differentiation (F_ST_ = 0.14–0.32, Table 4) was observed among all populations and New Zealand. This specific population showed the most distant genetic relatedness to the Canary Islands and North American populations and was equally close to the *P. multivora* populations from South Africa and Australia (F_ST_ = 0.14). The lowest but statistically significant differentiation (F_ST_ = 0.05) was observed between the South African and Australian populations. The European population did not show any statistically significant differentiation with the other populations but New Zealand.

**Table 4:** Pairwise F_ST_-values inferred from 10 SSR loci between the six *P. multivora* populations (South Africa, Australia, Canary Islands, Europe, New Zealand, and North America) analyzed in this study

Population (MLG)^**1**^	South Africa (34)	Australia (54)	Canary Islands (3)	Europe (7)	New Zealand (18)
Australia (54)	0.05^2^ (< 0.01)				
Canary Islands (3)	0.08 (0.46)	0.11 (0.26)			
Europe (7)	0.11 (0.05)	0.04 (0.38)	0.08 (0.62)		
New Zealand (18)	0.14 (< 0.01)	0.14 (< 0.01)	0.32 (0.01)	0.25 (< 0.01)	
North America (3)	0.17 (0.10)	0.07 (0.47)	0.35 (0.22)	0.06 (0.58)	0.31 (0.01)

^1^Number of multilocus genotypes in each population

^2^In brackets, the *P-*value resulting from a one-sided permutation test

**Fig. 1:**
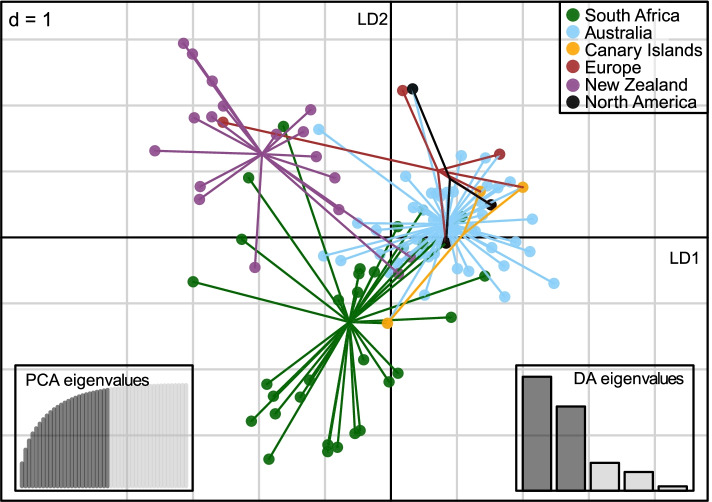
DAPC results were computed with the 119 MLGs detected in the six populations of *Phytophthora multivora* (South Africa, Australia, Canary Islands, Europe, New Zealand, and North America) analyzed. MLGs are color-coded by population, as indicated on the top of the scatterplot. Scatterplots represent the distribution of individual MLGs (symbols) along the first two linear discriminants. The cross-validated number of the principal components and selected linear discriminants used for the analysis is shown in dark color in the bar plots on the bottom left and right of the scatterplot, respectively

Clustering of *P. multivora* MLGs from different populations was retrieved with a discriminant analysis of principal components (DAPC, Fig. 1) defined from clone-corrected data (119 MLGs). According to the lowest root mean squared error and highest mean of successful reassignments with 1000 replicates (cross-validation), 25 of the 47 computed PCs were used to build discriminant functions. We observed a relatively distant and well-centered clustering of MLGs from the three most diverse populations (Fig. 1). Specifically, the New Zealand population discriminated along the first axis from the Australian population and along the second axis from the South African population. The other three less diverse populations (North America, Europe, and the Canary Islands) clustered within those centers and were mainly associated with Australian MLGs. Random MLGs from the European and Canary Islands populations were associated with the New Zealand and South African MLG clusters.

**Fig. 2:**
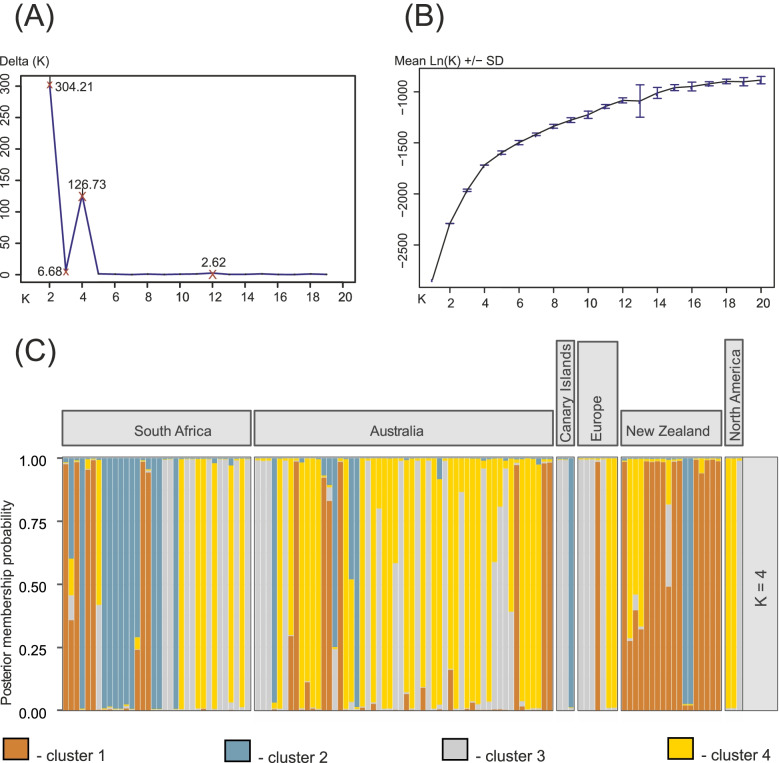
Results of the Structure analysis in the six populations of *Phytophthora multivora* (South Africa, Australia, Canary Islands, Europe, New Zealand, and North America). **A** Graph representing ΔK, with the four most considerable changes marked with a brown cross. **B** Graph representing the mean log-likelihood values (± standard deviation) for different numbers of clusters. The barplot represents the average estimated membership probability (y-axis) of an MLGs assigned to a specific cluster (indicated by specific color) among four clusters selected to describe the global population structure

Overall, multivariate discriminant analysis and the Structure Bayesian analysis showed congruent results. In the Structure analysis, considering alteration of assignments in admixed populations (Supplementary Fig. S3), the log-likelihood increasing up to 20 clusters (Fig. 2B) and the second-highest difference of the log-likelihood among different K (ΔK peak at K = 4, Fig. 2A), we assumed four clusters as reasonable to best describe the genetic structure in the global population of *P. multivora* (Fig. 2C). MLGs from South Africa were equally assigned to all four defined genetic clusters. The Australian population was co-dominated by MLGs assigned to the fourth (yellow) cluster followed by the third (grey) cluster. The remaining two clusters (blue and yellow) were least represented in this particular population. MLGs from New Zealand were mostly assigned to the first (orange) cluster with minor admixture of the fourth (yellow) cluster and two MLGs assigned to the second (blue) cluster. Finally, *P. multivora* populations from Europe and the Canary Islands were dominated by the third (grey cluster), whereas the North American population included two MLGs assigned to the fourth (yellow) and one to the third (grey) cluster. Noteworthy, no admixed MLGs (i.e. assigned partially to different clusters) were observed in these last three populations.

#### Likelihood population history

Considering the high diversity estimates, relatively low F_ST_ values, three-center clustering in the multivariate discriminant analysis, and diverse genetic population structure in the South African, Australian, and New Zealand populations, we assumed these populations were older and likely source of the other three populations. Indeed, populations of Europe, the Canary Islands, and North America showed a lower diversity and no or few private alleles and unique MLGs.

**Fig. 3:**
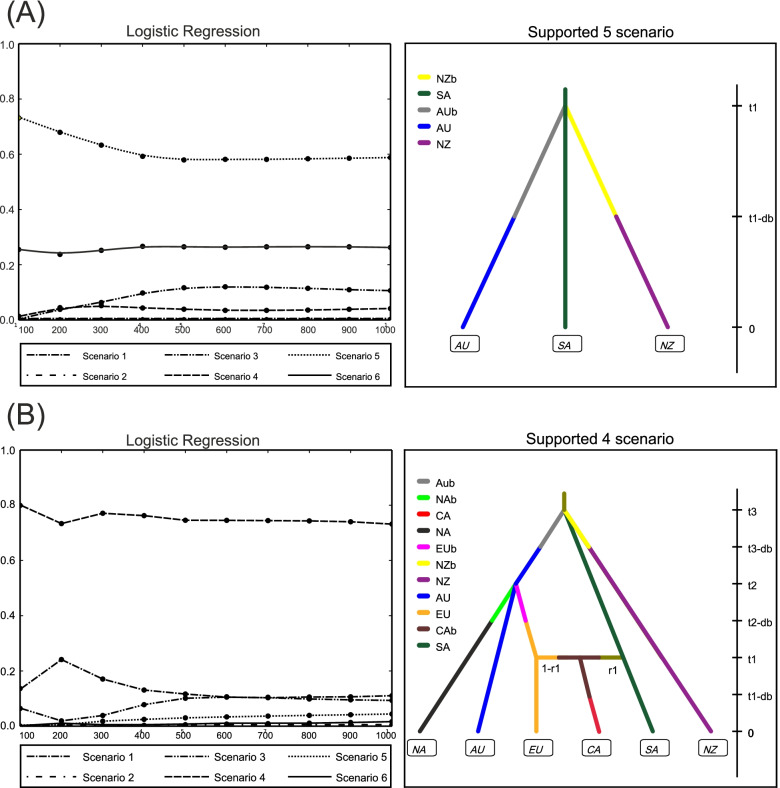
Graphic representation of the ABC analysis for reconstructing the global spread of *Phytophthora multivora*. **A** Identification of the native population. The graphic on the left side shows the relative posterior probabilities estimated from linear discriminants of the summary statistics inferred from 1000 of 6 × 10^6^ simulated datasets closest to observed of the six competing scenarios. The diagram on the right side shows the supported scenario, its demographic events, change in the effective population size (colored segment), and corresponding relative time (right vertical scale). As shown in the diagram, results were computed with the three most diverse populations: Australia - AU, South Africa - SA, and New Zealand -NZ. Abbreviation with an additional “b” indicates introduced and established populations with limited effective population sizes; db- time of establishing. **B** Global spread. The graphic on the left side shows the relative posterior probabilities estimated from linear discriminants of the summary statistics inferred from 1% of 10^6^ simulated datasets closest to the observed of the six competing scenarios. The diagram on the right side shows the supported scenario and its demographic events as described above. As shown in the diagram, results were computed with all six populations: Australia - AU, South Africa - SA, New Zealand - NZ, Canary Islands - CA, Europe - EU, North America – NA. Abbreviations with an additional “b” indicate introduced and establishing populations with limited effective population sizes; db- time of establishing. Time is not scaled on the schemes

Six different scenarios of the demographic history (see details on the competing scenario in Supplementary Notes 1, Fig. S4–5, Table S2–3) were tested with the Approximate Bayesian Computational (ABC) analysis to define the source population among the South African, Australian, and New Zealand populations. The highest posterior probabilities with non-overlapping 95% Cis, inferred from 500 simulated data closest to the observed using a direct approach (Supplementary Fig. S5, Table S2) and 1000 simulated data closest to the observed with a linear discriminant transformation (Fig. 3A left) of the summary statistic values were computed for the fifth scenario (Fig. 3A right, Supplementary Table S2). In this particular scenario, we assumed that at nominal time t1, two populations of a small effective size were introduced to New Zealand and Australia from the South African population. These two initial populations (AUb and NZb in Fig. 3A right) independently developed during the establishing time t1-db and resulted in the current populations (AU and NZ) at nominal time t0. We intentionally did not speculate about quantitative estimates of the time and effective population sizes of the historic populations retained in the ABC analysis. This is because of the difficulties in realistically addressing generation time or individuals’ number in oomycete species like *P. multivora*. Despite the possible uncertainty in the analysis of the specific quantitative results, the topology of our ABC analysis clearly showed the South African population is most likely the global source and place of origin of the species, and the two populations of New Zealand and Australia directly originated from it.

The second ABC step supported (Fig. 3B left) the fourth scenario (see details on the competing scenario in Supplementary Notes 1, Fig. S6–7, Table S4). This specific scenario suggested after the establishment of the Australian population, some MLGs were introduced to North America (specifically to the US) and Europe around the same time, nominally at time t2. These populations (NAb and EUb) of a limited effective size independently developed further, resulting in the populations sampled at time t0. After the establishment of the European population, at time t1 some MLGs were presumably introduced from both Europe and the native South African population to the Canary Islands, where they founded a relatively young and admixed population CAb that developed to the sampled population CA in Fig. 3B (left diagram).

#### Bayesian phylogeographic analyses

**Fig. 4:**
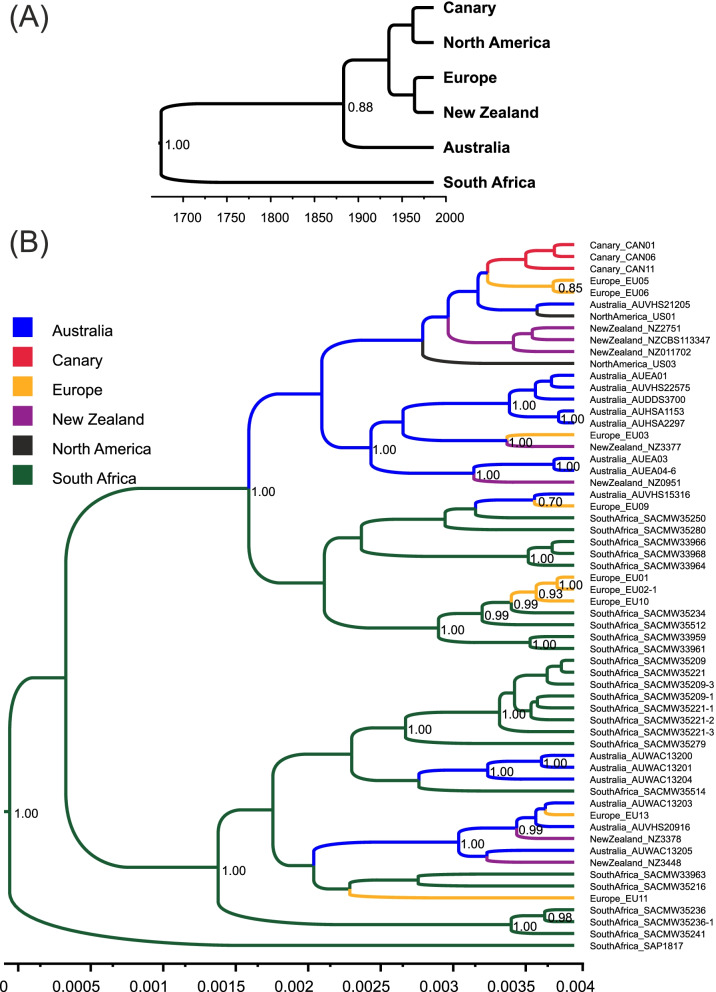
Maximum clade credibility trees of *Phytophthora multivora,* each was inferred from 9001 Bayesian trees that were sampled among 2 × 10^7^. Support of the phylogenetic clades with PP > 0.7 is shown in the nodes. **A** Generalized to population level phylogeny was reconstructed using sequences of three mitochondrial (*cox*I and *cox*IGS and NADHI) of 60 *P. multivora* samples from the six populations (South Africa, Australia, Canary Islands, Europe, New Zealand, and North America). Time is scaled according substitution rate 2.4 × 10^− 6^ per site and per year on the x-axis. **B** Phylogeny was reconstructed using sequences of three mitochondrial (*cox*I and *cox*IGS and NADHI), and three nuclear loci (ASF, ENOLASE, and HSP90) of 60 *P. multivora* samples from the six populations (South Africa, Australia, Canary Islands, Europe, New Zealand, and North America). Samples are named as follows: population, strain’s ID, and number when a cloned variant of a gene was present. Colors of branches indicate the most common location of the node with PP > 0.98 of the geographic origin of each clade

The best-fit models for each of the sequenced loci, specifically COI (concatenated *cox*I and *cox*IGS) and NADHI (mtDNA) as well as ASF, ENOLASE, HSP90 (nDNA), were as follows: HKY (Hasegawa-Kishino-Yano) for both mtDNA loci, JC69 (Jukes-Cantor) for the ASF locus and TN93 (Tamura-Nei) for the other two nDNA loci. Both Bayesian coalescent analysis we conducted, i.e. StarBeast with multilocus mitochondrial data and phylogeographic MASCOT with three nuclear and three mitochondrial loci, showed with high posterior probabilities that MLGs from South Africa are likely representing an ancestral lineage to the current global population of *P. multivora* (Fig. 4A, B). The genealogy reconstructed with the StarBeast method and scaled to time according to the 2.4 × 10^− 6^ per site and per year [40] substitution rate for mitochondrial genome showed that divergence of South African and Australian populations (Fig. 4A) might have occurred 300–400 years ago, while the divergence of Australian and others populations analyzed in the study started at the end of the nineteenth century. Results of MASCOT indicated South Africa as the most common location of the root node with a posterior probability (PP) of 0.996 against < 0.004 for any other location (Fig. 4B). The maximum-clade-credibility tree discriminated further MLGs into two major clades, for both of which South Africa was determined as ancestral location (PP 0.99 vs. < 0.011 for other populations). In the first clade from above (Fig. 4B), the Australian lineage diverged from the South African sister clade. This new (blue) clade harbored most MLGs from New Zealand, North America, the Canary Islands and Europe and, with high posterior probability support (0.99 vs < 0.002 for others populations), had Australia as the source location. However, three MLGs from Europe were more closely related to South African MLGs (Fig. 4B). In addition, we observed high estimates of migration from South Africa to Europe (Supplementary Table S5); suggesting a direct origin of part of the European *P. multivora* population from the ancestral South African population. A few Australian MLGs did not cluster within the major (blue) Australian clade, and might be the consequence of repeated introductions of *P. multivora* to Australia, mainly from South Africa.

## Discussion

Our analyses shed light on the population diversity, reproductive biology, and invasion history of *Phytophthora multivora*. We detected substantial genotypic diversity with polymorphic SSR markers, i.e. 119 MLGs among the 306 isolates analyzed. Nevertheless, only two MLGs, both occurring in Australia, were heterozygous. The homothallic mating system of *P. multivora* could explain the lack of heterozygotes. Similar extremely low heterozygosity was observed in populations of other homothallic *Phytophthora* species, e.g. in *P. sojae* [41] and *P. plurivora* [23]. Homothallism implies self-fertilization during sexual reproduction (i.e. oospore formation), which leads to extensive inbreeding and the reduction of heterozygosity in a population [42]. However, homothallic species can sometimes outcross, and heterothallic species can sometimes self-fertilize [43]. We also detected positive and significant indexes of association (I_A_ and rd), indicating nonrandom association of loci. Among several reasons for the deviation from the random association of gametes (panmixia), the most common for oomycetes or fungi are asexual and clonal reproduction [44]. Indeed, *P. multivora,* like many other species in the genus, grows clonally and produces asexual sporangia, releasing zoospores. Zoospores dispersed through the soil water are the main infective propagules [45, 46]. However, the predominance of asexual reproduction would also lead to lower genotypic diversity and higher heterozygosity [44]. Hence, the extreme lack of heterozygosity, substantial MLG diversity, and deviation from panmixia, suggest *P. multivora* readily propagates both from oospores through homothallic self-fertilization and asexual zoospores, but gene flow among studied populations is restricted*.*

Our study revealed relatively high genetic diversity with both genetic markers in the three *P. multivora* populations, specifically in South Africa, Australia, and New Zealand. Correspondingly, all MLGs clustered around three centers associated with these geographic locations in the multivariate non-parametric analysis (DAPC in Fig. 1), suggesting those populations are older than others and are most likely already well established. In contrast, the other populations of North America, Europe, and the Canary Islands were substantially less diverse. A few MLGs from these populations are not unique, but occurred in three other populations, and clustered predominantly around the Australian center, suggesting their secondary origin.

*Phytophthora multivora* became known worldwide due to its distribution and devastating effect on woody plants in Western Australia [12, 24]. Later, this species was discovered widespread in soil, streams, and the rhizosphere of asymptomatic vegetation in South Africa [25]. The same study revealed high genetic diversity of the South African population, similar to the population of West Australia. However, in South Africa, unlike in Australia, *P. multivora* was not associated with any disease outbreaks or extensive plant mortality [25, 47–49], suggesting a long-term co-evolution between native tree species and the pathogen. The species was also retrieved from waterways and soil of disease foci in New Zealand. However, its ecological role is still unclear [49–51]. In the current study, the genetic diversity of *P. multivora* was slightly lower in New Zealand than in South Africa and Australia. Noteworthy, the New Zealand population showed the highest number of unique genotypes and highest F_ST_ values, indicating the most distant relatedness to other populations in the world. This might reflect an ancient introduction from a more diverse source population and then an isolated evolution of the New Zealand population. Such an introduction might be a consequence of the uncontrolled but considerable intra-regional trades of woody plants and seedlings between New Zealand and Australia, or/and intercontinental import directly from South Africa during colonization of both Australia and New Zealand. During colonization of Australia and New Zealand, Capetown was a port of call on the voyage from Europe [52]. The spread of many known forest pests and pathogens was predominantly assisted by human activity under fast globalization [53]. For example, *P. ramorum* and *Cryphonectria parasitica* were introduced to North America and Europe through nursery stock import [54, 55], *P. cinnamomi* invasion was associated with the trade of agricultural commodities [38], the trade of wooden logs contributed to Dutch elm disease caused by *Ophiostoma ulmi* and *O. novo-ulmi* [53, 56], and the use of wooden packages for long-distance transportation is responsible for the global spread of the Asian longhorned beetle (*Anoplophora glabripennis*) [57]. Furthermore, even traded forest seeds can be a source of pathogens [58, 59]. The upsurge of invasions raises the urgent need to consider global quarantine management [60].

The global invasion of *P. multivora* most likely commenced from South Africa. We detected the most complex genetic structure in this particular population, i.e. local MLGs were assigned to all four genetic clusters defined in STRUCTURE analysis in nearly equal proportion (Fig. 2), congruent with high diversity estimates of summary statistics. The Australian and New Zealand populations showed co-dominant clusters, suggesting genetic diversity was only partially preserved in those populations over time. These observations were confirmed by the approximate Bayesian computation analysis (ABC) and suggested the Australian and New Zealand populations originated from South Africa and experienced an establishing time of limited effective sizes. During the lag invasion phase, some specific genotypes may go lost due to natural selection or by chance (drift) in a population, while others may successfully spread. Alternatively, only a few specific genotypes were initially introduced in each of two populations, or as suggested by ABC analyses, there was no direct introduction from South Africa to New Zealand but via the bridgehead through Australia. In this case, most likely, the limited number of genotypes arrived in New Zealand from Australia and established a new, less diverse population. This invasion scenario is supported by the relatively lower diversity estimates and the presence of only two genetic clusters with non-admixed MLGs (Fig. 2) in New Zealand compared to the presence of all four in Australia. Similar to our study on *P. multivora*, the bridgehead effect was reported for many globally dispersed invasive pests and pathogens of plants [61–65]. The bridgehead effect describes the scenario where an invasive pest is first established in a new area after which secondary spread occurs, leading to the foundation of new invasive populations [63]. Frequently, due to global human trades and movement, the step population is as distant as a different continent.

Overall, the results obtained with Bayesian coalescent analyses of both nuclear and mitochondrial sequences confirmed our findings in population structure and ABC history analysis of 10 SSR genetic markers. In particular, both analyses are in accordance with South Africa being the center of origin of *P. multivora*, and Australia being the primary distributor of the species worldwide. Multilocus coalescent genealogy based on mitochondrial loci and scaled to time showed that South African and Australian populations might had diverged 300–400 years ago, which corresponds to the period of European exploration of Australia and Dutch colonization of South Africa. The secondary spread of *P. multivora* from the bridgehead population in Australia and the consequent establishment of new populations throughout the world might had started at the end of the nineteenth century during intensification of global trade and travels, which facilitate introduction of alien pathogens in new ecosystems [66]. Hence, *P. multivora* is another plant pathogen first described outside its center of origin due to the extensive damage caused on naïve species in the introduced range. Likewise, *P. infestans*, the responsible for the Irish potato famine (1845), originated most likely from Mexico [40]. Similarly, *C. parasitica*, the causal agent of chestnut blight, was first detected in North America and Europe and not in its native range in Eastern Asia [55], as it happened for *Hymenoscyphus fraxineus* (synonym: *H. pseudoalbidus*), the ascomycete responsible for the epidemic of ash dieback in Europe [67].

**Fig. 5:**
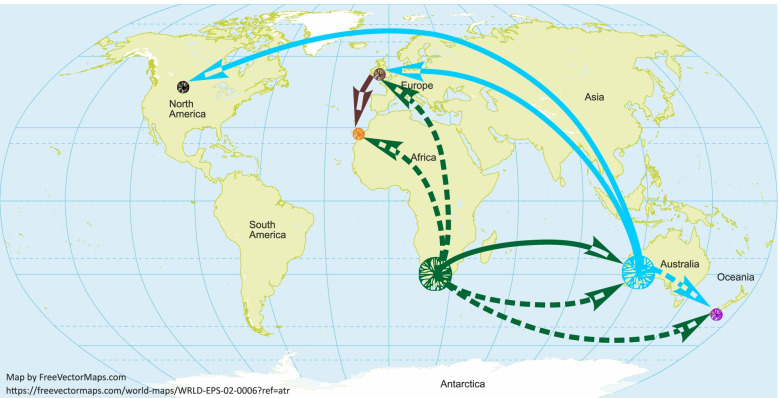
Inferred global invasion history of the plant pathogen *Phytophthora multivora*. Solid lines indicate path confirmed by both ABC and MASCOT analysis; dashed lines show pathways inferred in one of the performed analyses

Besides confirming the South African origin of *P. multivora*, our analyses of a large collection of isolates from six geographic populations suggest a complex spread of this species throughout the world with possible multiple introductions to specific continents (Fig. 5). In particular, some genotypes were directly introduced from South Africa to Europe in addition to those introduced from Australia. The resulted genealogy indicates paraphyletic groups of Australian and South African genotypes cluster together, suggesting possible multiple introductions of *P. multivora* to Australia from South Africa. Both coalescent analyses, ABC and MASCOT, are in accordance that the North American population originated from Australia. While genotypes from the Canary Islands have a common ancestor with European isolates, they altogether might have Australian descent. Although there is no confirmation found in the phylogenetic coalescent analysis, that some *P. multivora* genotypes might had been introduced directly from South Africa to the Canary Islands, as suggested by the ABC analysis. Thus, the European population was most likely a secondary bridgehead for the invasion of the Canary Islands.

## Conclusions

To summarize, our genetic analysis of the global population revealed the invasive history of *Phytophthora multivora,* emerging plant pathogen, most likely started in South Africa, followed by its introduction to Australia, and then spread worldwide from there. The conclusions were made based on a large collection of isolates from six populations (South Africa, Australia, Canary Islands, Europe, New Zealand, and North America) from geographic regions where *P. multivora* is currently known as widely distributed and abundant, or/and where it has a severe phytopathological impact. In future, new *P. multivora* populations might be discovered and our results will have to be updated.

## Methods

### Phytophthora multivora isolates

We obtained 335 isolates of *P. multivora* from six distinct geographic locations: North America, Europe, South Africa, Australia, the Canary Islands, and New Zealand (Table S1).

### Single sequences repeat (SSR) genotyping

Genomic DNA was extracted from the selected pure cultures of *P. multivora* using the DNeasy Plant Mini kit (Qiagen, Hilden, Germany) according to the manufacturer’s protocol. All isolates were then genotyped at 10 microsatellite loci (PmMS02, PmMS04, PmMS06, PmMS07, PmMS08, PmMS10, PmMS12, PmMS14, PmMS18, and PmMS24) that were previously developed by [68]. All loci were PCR amplified using the following program: initial denaturation at 95 °C for 5 min; followed by 27 cycles of 95 °C for 30 s, 59 °C for 90 s, and 72 °C for 30 s; and a final extension of 60 °C for 30 min. The 10 microsatellite loci were amplified in three multiplex PCR reactions targeting each three to four loci (reaction 1: PmMS02, PmMS04, PmMS06, and PmMS07; reaction 2: PmMS08, PmMS10, and PmMS12; reaction 3: PmMS14, PmMS18, and PmMS24). All reactions were performed in 10 μL volumes containing final concentrations of 1x Type-it Multiplex PCR Master Mix (Qiagen) and 0.2 μM of each primer and 1 μL DNA. Forward primers were labeled at the 5′-end with a fluorescent dye (FAM: PmMS02-F, PmMS06-F, and PmMS12-f; PET: PmMS04-F, PmMS18-F, and PmMS24-F; NED: PmMS07-F, PmMS10-F, and PmMS14-F; VIC: PmMS08-F), and amplicons were run on an ABI 3130 Genetic Analyzer using GeneScan 500 LIZ (Applied Biosystems, Carlsbad, CA, USA) as an internal size standard. Allele sizes were scored with GeneMapper Software 5 (Applied Biosystems).

### Amplification of mitochondrial and nuclear loci

Six gene regions, 3 nuclear and 3 mitochondrial, were sequenced for 94 isolates of *P. multivora* (Table S1); 22 from South Africa, 30 from Western Australia, 3 from eastern Australia, 7 from the Canary Islands, 12 from Europe, 12 from New Zealand and 8 from the United States.

Genomic DNA was extracted from isolates as described previously [69]. For products to be cloned, GoTaq Hot Start Polymerase (Promega, Madison, USA) and buffer were used. Six gene regions were amplified; the mitochondrial intergenic spacer (*cox*IGS) between cytochrome oxidase 2 and cytochrome oxidase 1 [70], and the partial coding sequence for the cytochrome oxidase 1 (*cox*I) [70], NADH dehydrogenase subunit 1 (NADHI) [71], *Enolase* (ENOLASE) [72], Heat shock protein 90 (HSP90) [72], and the anti-silencing factor (ASF)-like gene (ASF) [70]. The reaction mixtures and cycling conditions for the amplification were as described previously in the original publications, except that 2 μL of 1:10 diluted genomic DNA was used as a template. Products were cloned if additivity was observed in the initial sequence. These amplicons were cloned into a bacterial plasmid vector, pGEM®-T Easy Vector System, as described previously [47], and 6–10 colonies were sequenced for each. The clean-up of amplicons using Sephadex and sequencing as described previously [73]. All sequences derived in this study are available from Data Dryad (https://datadryad.org).

### Data analysis

We considered samples collected from each of the six geographically distinct locations as a single population of *P. multivora*. We used 10 SSR loci to study current population genetic diversity, structure, and demographic history of the global spread of *P. multivora* with Approximate Bayesian Computation (ABC). The SSR is generally considered neutral genetic markers [74] and therefore appropriate for stated research questions. Furthermore, we reconstructed the evolution of the *P. multivora* global population with Bayesian coalescent analysis using sequences of the three nuclear and three mitochondrial loci. In order to remove a putative clonal effect on the genetic structure, only one representative of each multilocus genotype (MLG) per population was considered.

#### Genetic diversity

Summary statistics on MLG, diversity indexes [75] and expected heterozygosity [76] were determined using the R-package *poppr* v 2.9.0 [77]. Allelic richness (Ar) per SSR locus and observed heterozygosity were estimated using the package Hierfstat v 0.5–7 [78]. The deviation from Hardy-Weinberg equilibrium [HWE, 79] was estimated using Arlequin 3.5.2.1 [79]. Pairwise linkage disequilibrium between loci was tested with the log-likelihood ratio using a Markov chain algorithm (default parameters), as implemented in the web version of Genepop 4.2 [80]. The statistical significance of LD was inferred using 1000 permutations and a sequential Bonferroni correction with α = 0.05. Genetic differentiation among populations was assessed by calculating pairwise *F*_ST_-values [81] and corresponding *P-*values (α = 0.05) with Arlequin 3.5.2.1. Multilocus linkage disequilibrium was evaluated based on the *P-*values from one-sided permutation tests with the R-package *poppr* v 2.9.0 for the indices of association I_A_ and rD (Agapow & Burt 2001).

#### Population structure

Genetic kinship of the *P. multivora* isolates recovered from different continents was examined using a multivariate clustering method, i.e. discriminant analysis of principle components (DAPC), implemented in the R-package *adegenet* [82]. First, multilocus genetic data were transformed into principal components (PCs), and the optimal number of PCs was determined with cross-validation [82]. Then *P. multivora* isolates with correspondent PCs were plotted along with the first two discriminant functions.

The genetic structure of the *P. multivora* global population with six assigned populations corresponding to the isolate’s geographic origin (i.e. North America, Europe, South Africa, Australia, the Canary Islands, and New Zealand) was studied with the Bayesian model-based cluster analysis, as implemented in STRUCTURE v 2.3.4. The isolates were probabilistically assigned to genetic clusters using allele frequencies at each SSR locus. We used sampling locations of the populations as prior geographic information (LOCPRIOR = 1 option) and the admixture ancestral model with correlated allele frequencies. Analyses were run with 200,000 burn-in iterations, followed by the same number of iterations for Markov chain Monte Carlo (MCMC) in 10 independent runs for each number of clusters (K) from 1 to 20. The most likely K was determined, as suggested in [83], by (1) considering the maximal mean and small standard deviation of the posterior probability of K among runs [84], (2) applying ΔK methods [85], using Structure Harvester [86], and (3) analyzing the alterations of individual assignment probabilities with increasing K (i.e. whether additional clusters were represented with a high probability by at least one specimen or whether probabilities rather were portioned among several individuals). Average assignment probabilities of specimens to the genetic clusters were computed with Clumpp 1.1.2 [87] using the greedy algorithm for K ≥ 10 and visualized using R graphic functions.

#### History of spread

The demographic history of the *P. multivora* spread among continents and the Canary Islands and New Zealand were investigated using a coalescent approximate Bayesian computation approach implemented in DIYABC v.2.1.0 [88]. The demographic scenario that best explained the observed genetic diversity in populations was inferred from two analysis steps (for details and prior population parameters, see Supplementary information). First, the six scenarios of the global origin of *P. multivora* were tested using the three most genetically diverse, and thus most likely oldest populations (see Results); populations from South Africa, Australia, and New Zealand. Then, a range of sequential runs of determining the most probable alternative scenario was performed by adding one of the remaining less diverse populations; European, North American, and the Canary Islands’ population (data not shown). Finally, considering population genetics results and assessments of intermediate evaluations of the alternative scenario for three minor populations, six most likely scenarios of the global distribution of *P. multivora* were hypothesized and tested. ABC analysis was conducted following [89], and included the following steps: 1) assume realistic competing scenarios considering structure, F_ST_ ratio between sampled populations, and field observations; 2) simulate 1 × 10^6^ pseudo-observed datasets (PODs) for each scenario and compute correspondent summary statistics; 3) evaluate posterior probabilities of each scenario on 1000 PODs with the closest summary statistic to the observed dataset and identify the best scenario in 95% confidence interval; 4) assess the confidence level of the chosen scenario as the proportion of times this scenario was falsely rejected (type-I error) or accepted (type-II error); 5) evaluate the goodness-of-fit of the selected scenario to the data.

#### Phylogeography

In order to study the phylogeographic evolution of the *P. multivora* global population, we used sequences of three mitochondrial loci and three nuclear loci for each specimen of the studied geographic locations. The alignments of each molecular locus were done using the ClustalW method, then the substitution model that best fitted to the locus data based on the lowest BIC scores (Bayesian Information Criterion) was selected using MEGA7 software [90]. For each gene, only haplotypes were used to determine nucleotide and sequence diversity estimates as implemented in DNASP v. 6 [91].

Bayesian inferences about the evolution of the *P. multivora* global population were conducted by sampling trees with BEAST v2.6.2 package [92, 93]. MCMC runs with 2 × 10^7^ iterations were carried out. The effective sample size estimates were assessed in Tracer v1.7.1 [94]. The substitution model that best fit according to the lowest BIC was set for each locus. A strict molecular clock model was used. We estimated genealogical tree and time of coalescent events for six populations (i.e. North America, Europe, South Africa, Australia, the Canary Islands, and New Zealand) of 60 *P. multivora* specimens in total, using their mitochondrial multilocus data, specifically COI (concatenated *cox*I and *cox*IGS) and NADH1, following the statistical methodology implemented in StarBeast [95, 96]. In order to compute time of coalescent events, we used a substitution rate of 2.4 × 10^− 6^ per site and per year, which was previously estimated for mitochondrial genomes of *P. infestans* [40]. Given the lack of recombination and relatively conservative evolution of the mitochondrial genome [41], we assumed this substitution rate to be applicable within the genus *Phytophthora*. We defined ancestral geographic location states following Marginal Approximation of the Structured Coalescent method [97, 98]. Specifically, we reconstructed the MASCOT tree for 60 specimens of *P. multivora* (Supplementary Table S1) from six geographically distant locations, using multilocus sequence data with nuclear genes (i.e. ASF, ENOLASE*,* and HSP90) and the three mitochondrial genes mentioned above. The mutation rate was set as a constant 1.0 and the estimation of branch lengths was calculated in substitutions per site. The summarizing trees for the phylogeographic origin of the *P. multivora* populations were executed using the location of origin as discrete states. The substitution rate and the substitution model that best fit according to the lowest BIC were set for each locus. The default priors for the StarBeast and the MASCOT trees with Log Normal options and exponential population models were generated using Beauti v2.6.6 [92, 93].. Maximum clade credibility consensus trees with mean node heights and 0.5 posterior probability limit were generated using TreeAnnotator v2.6.3 [92] and then visualized using FigTree v1.4.4.

## Supplementary Information

**Additional file 1.**

## Data Availability

The datasets analysed during the current study are available in the Data Dryad repository by next links: 10.5061/dryad.gxd2547nr

## Abbreviations

DAPC: Discriminant analysis of principal components
MASCOT: Marginal approximation of the structured coalescent analysis
MLG: Multilocus genotypes
PP: Posterior probability
SSR: Single sequences repeats
