# Supplementary material for: Global invasion history of the emerging plant pathogen Phytophthora multivora
Source: BMC Genomics. 2022 Feb 22;23:153. doi: 10.1186/s12864-022-08363-5 (PMC8862219; doi:10.1186/s12864-022-08363-5)
Supplement: Supplementary file 1 — Additional file 1. [file 12864_2022_8363_MOESM1_ESM.pdf]

Supplementary information

Table S1. *Phytophthora multivora* isolates used in this study

No.	Isolate	Continent Islands	Country	Region	Host or substrate	Year	SSR_ID	Seq_ID
1	CMW35215	Africa	South Africa	Kwa Zulu Natal, Umtamvuna	Forest soil	2010	SA_CMW35215	-
2	CMW35217	Africa	South Africa	Kwa Zulu Natal, Umtamvuna	Forest soil	2010	SA_CMW35217	-
3	CMW35218	Africa	South Africa	Kwa Zulu Natal, Umtamvuna	Forest soil	2010	SA_CMW35218	-
4	CMW35220	Africa	South Africa	Kwa Zulu Natal, Umtamvuna	Forest soil	2010	SA_CMW35220	-
5	CMW35222	Africa	South Africa	Kwa Zulu Natal, Umtamvuna	Forest soil	2010	SA_CMW35222	-
6	CMW35237	Africa	South Africa	Kwa Zulu Natal, Ingeli Forest	Forest soil	2010	SA_CMW35237	-
7	CMW35239	Africa	South Africa	Kwa Zulu Natal, Ingeli Forest	Forest soil	2010	SA_CMW35239	-
8	CMW35243	Africa	South Africa	Kwa Zulu Natal, Ingeli Forest	Forest soil	2010	SA_CMW35243	-
9	CMW35245	Africa	South Africa	Kwa Zulu Natal, Ingeli Forest	Forest soil	2010	SA_CMW35245	-
10	CMW35249	Africa	South Africa	Kwa Zulu Natal, Ingeli Forest	Forest soil	2010	SA_CMW35249	-
11	CMW35251	Africa	South Africa	Kwa Zulu Natal, Ingeli Forest	Forest soil	2010	SA_CMW35251	-
12	CMW35253	Africa	South Africa	Kwa Zulu Natal, Ingeli Forest	Forest soil	2010	SA_CMW35253	-
13	CMW35255	Africa	South Africa	Kwa Zulu Natal, Ingeli Forest	Forest soil	2010	SA_CMW35255	-
14	CMW35320	Africa	South Africa	Kwa Zulu Natal,	*Bauhiuia variegata*	2010	SA_CMW35320	-
15	CMW35322	Africa	South Africa	Kwa Zulu Natal,	*Magnolia soulangiana*	2010	SA_CMW35322	-
16	CMW35324	Africa	South Africa	Kwa Zulu Natal,	*Camellia* spp.	2010	SA_CMW35324	-
17	CMW35326	Africa	South Africa	Kwa Zulu Natal,	*Camellia* spp.	2010	SA_CMW35326	-
18	CMW35330	Africa	South Africa	Kwa Zulu Natal,	Forest soil	2010	SA_CMW35330	-
19	CMW35331	Africa	South Africa	Kwa Zulu Natal,	Forest soil	2010	SA_CMW35331	-
20	CMW35332	Africa	South Africa	Kwa Zulu Natal,	Forest soil	2010	SA_CMW35332	-
21	CMW35513	Africa	South Africa	Kwa Zulu Natal,	*Liriodendron tulipifera*	2010	SA_CMW35513	-
22	CMW35959	Africa	South Africa	Mpumulunga, Lydenburg, Buffelskloof NR	Water	2010	SA_CMW35959	-
23	P1821	Africa	South Africa	-	*Ocotea bullata*	2010	SA_P1821	-
24	CMW33963	Africa	South Africa	Transvaal, Buckelskloof	Forest soil	2010	SA_CMW33963	CMW33963
25	CMW33964	Africa	South Africa	Western Cape, Jonkershoek, Nature Reserve	Forest soil	2010	SA_CMW33964	CMW33964
26	CMW33967	Africa	South Africa	Western Cape, Bettys Bay Harold Potter NBG	*Rapanea* spp.	2010	SA_CMW33967	CMW33967
27	CMW35209	Africa	South Africa	Kwa Zulu Natal, Umtamvuna	*Cryptocarya latifolia*	2010	SA_CMW35209	CMW35209
28	CMW35209	Africa	South Africa	Kwa Zulu Natal, Umtamvuna	*Cryptocarya latifolia*	2010	-	CMW35209-1
29	CMW35209	Africa	South Africa	Kwa Zulu Natal, Umtamvuna	*Cryptocarya latifolia*	2010	-	CMW35209-3
30	CMW35221	Africa	South Africa	Kwa Zulu Natal, Umtamvuna	Forest soil	2010	SA_CMW35221	CMW35221
31	CMW35221	Africa	South Africa	Kwa Zulu Natal, Umtamvuna	Forest soil	2010	-	CMW35221-1
32	CMW35221	Africa	South Africa	Kwa Zulu Natal, Umtamvuna	Forest soil	2010	-	CMW35221-2
33	CMW35221	Africa	South Africa	Kwa Zulu Natal, Umtamvuna	Forest soil	2010	-	CMW35221-3
34	CMW35236	Africa	South Africa	Kwa Zulu Natal, Ingeli Forest	Forest soil	2010	SA_CMW35236	CMW35236
35	CMW35236	Africa	South Africa	Kwa Zulu Natal, Ingeli Forest	Forest soil	2010	-	CMW35236-1
36	CMW35236	Africa	South Africa	Kwa Zulu Natal, Ingeli Forest	Forest soil	2010	-	CMW35236-2
37	CMW35241	Africa	South Africa	Kwa Zulu Natal, Ingeli Forest	Forest soil	2010	SA_CMW35241	CMW35241
38	CMW35279	Africa	South Africa	Kwa Zulu Natal,	Forest soil	2010	SA_CMW35279	CMW35279
39	CMW35514	Africa	South Africa	Kwa Zulu Natal,	*Liriodendron tulipifera*	2010	SA_CMW35514	CMW35514
40	P1817	Africa	South Africa	RSA, Western Cape	*Medicago sativa*	2011	SA_P1817	P1817
41	P1817	Africa	South Africa	RSA, Western Cape	*Medicago sativa*	2011	-	P1817-2
42	P1818	Africa	South Africa	RSA, Western Cape	*Medicago sativa*	2011	SA_P1818	P1818
43	CMW35216	Africa	South Africa	Kwa Zulu Natal, Umtamvuna	Forest soil	2010	SA_CMW35216	CMW35216
44	CMW33966	Africa	South Africa	Western Cape, Bettys Bay Harold Potter NBG	*Rapanea* spp.	2010	SA_CMW33966	CMW33966
45	CMW33968	Africa	South Africa	Western Cape, Bettys Bay Harold Potter NBG	*Rapanea* spp.	2010	SA_CMW33968	CMW33968
46	CMW35250	Africa	South Africa	Kwa Zulu Natal, Ingeli Forest	Forest soil	2010	SA_CMW35250	CMW35250
47	CMW35280	Africa	South Africa	Kwa Zulu Natal,	*Prunus* spp.	2010	SA_CMW35280	CMW35280
48	CMW33959	Africa	South Africa	Mpumulunga, Lydenburg, Buffelskloof NR	*Heteropyxis canescens*	2010	-	CMW33959
49	CMW33959	Africa	South Africa	Mpumulunga, Lydenburg, Buffelskloof NR	*Heteropyxis canescens*	2010	-	CMW33959-1
50	CMW33959	Africa	South Africa	Mpumulunga, Lydenburg, Buffelskloof NR	*Heteropyxis canescens*	2010	-	CMW33959-2
51	CMW33961	Africa	South Africa	Mpumulunga, Schagen, Near Sudwala Caves	Forest soil	2010	SA_CMW33961	CMW33961
52	CMW35234	Africa	South Africa	Kwa Zulu Natal, Ingeli Forest	Water	2010	SA_CMW35234	CMW35234
53	CMW35235	Africa	South Africa	Kwa Zulu Natal, Ingeli Forest	Water	2010	SA_CMW35235	CMW35235
54	CMW35512	Africa	South Africa	Kwa Zulu Natal, Pietermaritzburg, NBG	*Camellia* spp.	2010	SA_CMW35512	CMW35512
55	DDS1160	Australia	Australia	WA, Ellis Creek SF	*Banksia grandis*	1986	AuWe_DDS1160	-
56	DDS1177	Australia	Australia	WA, Leeuwin-Naturaliste NP	*Dryandra sessilis*	1986	AuWe_DDS1177	-
57	DDS3368	Australia	Australia	WA, Eneabba	*Gastrolobium spinosum*	1990	AuWe_DDS3368	-
58	DDS3411	Australia	Australia	WA, Donnelly SF	*Patersonia* spp.	1991	AuWe_DDS3411	-
59	DDS3440	Australia	Australia	WA, Lake Muir SF	Forest soil	1992	AuWe_DDS3440	-
60	DDS3445	Australia	Australia	WA, Lake Muir SF	*Patersonia* spp.	1992	AuWe_DDS3445	-
61	DDS3458	Australia	Australia	WA, Perth	*Conospermum* spp.	1992	AuWe_DDS3458	-
62	DDS3472	Australia	Australia	WA, Lake Muir SF	*Patersonia* spp.	1992	AuWe_DDS3472	-
63	DDS3480	Australia	Australia	WA, Perth	*Lambertia echinata*	1992	AuWe_DDS3480	-
64	DDS3566	Australia	Australia	WA, Mount Frankland NP	*Patersonia* spp.	1993	AuWe_DDS3566	-

65	DDS3571	Australia	Australia	WA, Fitzgerald River NP	*Banksia attenuata*	1993	AuWe_DDS3571	-	
66	DDS3613	Australia	Australia	WA, Donnelly SF	*Patersonia* spp.	1994	AuWe_DDS3613	-	
67	DDS3623	Australia	Australia	WA, North Donnelly SF	*Patersonia* spp.	1994	AuWe_DDS3623	-	
68	DDS3634	Australia	Australia	-	*Banksia attenuata*	1994	AuWe_DDS3634	-	
69	DDS3661	Australia	Australia	WA, Donnelly SF	*Patersonia* spp.	1994	AuWe_DDS3661	-	
70	DDS3688	Australia	Australia	WA, Solai FB	Forest soil	1995	AuWe_DDS3688	-	
71	DDS3690	Australia	Australia	WA, Solai FB	Forest soil	1995	AuWe_DDS3690	-	
72	DDS3691	Australia	Australia	WA, Solai FB	Forest soil	1995	AuWe_DDS3691	-	
73	DDS3692	Australia	Australia	WA, Solai FB	Forest soil	1995	AuWe_DDS3692	-	
74	DDS3693	Australia	Australia	WA, Solai FB	Forest soil	1995	AuWe_DDS3693	-	
75	DDS3695	Australia	Australia	WA, Solai FB	Forest soil	1995	AuWe_DDS3695	-	
76	DDS3698	Australia	Australia	WA, Solai FB	Forest soil	1995	AuWe_DDS3698	-	
77	DDS3701	Australia	Australia	WA, Yeagarup	Forest soil	1995	AuWe_DDS3701	-	
78	DDS3702	Australia	Australia	WA, Yeagarup	Forest soil	1995	AuWe_DDS3702	-	
79	DDS3704	Australia	Australia	WA, Yeagarup	Forest soil	1995	AuWe_DDS3704	-	
80	DDS3706	Australia	Australia	WA, Warren SF	Forest soil	1995	AuWe_DDS3706	-	
81	DDS3708	Australia	Australia	WA, Granite Peaks SF	Forest soil	1995	AuWe_DDS3708	-	
82	DDS3715	Australia	Australia	WA, Mount Frankland NP	Forest soil	1995	AuWe_DDS3715	-	
83	DDS3716	Australia	Australia	WA, Mount Frankland NP	Forest soil	1995	AuWe_DDS3716	-	
84	DDS3734	Australia	Australia	WA, Mount Frankland NP	Forest soil	1995	AuWe_DDS3734	-	
85	DDS3749	Australia	Australia	WA, Warren SF	Forest soil	1995	AuWe_DDS3749	-	
86	DDS3811	Australia	Australia	WA, North Donnelly SF	Patersonia	1996	AuWe_DDS3811	-	
87	SLPA64	Australia	Australia	VIC. Sugarloaf	Forest soil	2008	AuVi_SLPA64	-	
88	VHA3414	Australia	Australia	WA, Albany	Forest soil	1991	AuWe_VHA3414	-	
89	VHS10041	Australia	Australia	WA, Tone SF	Patersonia	2001	AuWe_VHS10041	-	
90	VHS12170	Australia	Australia	WA, Huntley Mine	*Eucalyptus marginata*	2003	AuWe_VHS12170	-	
91	VHS13703	Australia	Australia	WA, Banksiadale SF	*Eucalyptus marginata*	2004	AuWe_VHS13703	-	
92	VHS13788	Australia	Australia	WA, Banksiadale SF	*Eucalyptus marginata*	2004	AuWe_VHS13788	-	
93	VHS14231	Australia	Australia	WA, Warren SF	*Leucopogon verticillatus*	2005	AuWe_VHS14231	-	
94	VHS14402	Australia	Australia	WA, Banksiadale SF	*Eucalyptus marginata*	2005	AuWe_VHS14402	-	
95	VHS14645	Australia	Australia	WA, Banksiadale SF	Forest soil	2005	AuWe_VHS14645	-	
96	VHS14716	Australia	Australia	WA, Lake Muir SF	*Patersonia* spp.	2005	AuWe_VHS14716	-	
97	VHS14926	Australia	Australia	WA, Banksiadale SF	*Eucalyptus marginata*	2005	AuWe_VHS14926	-	
98	VHS14993	Australia	Australia	-	Forest soil	2006	AuWe_VHS14993	-	
99	VHS15571	Australia	Australia	WA, Perth	*Banksia grandis*	2006	AuWe_VHS15571	-	
100	VHS15636	Australia	Australia	WA, Warren SF	*Patersonia* spp.	2006	AuWe_VHS15636	-	
101	VHS15860	Australia	Australia	WA, Wungong	*Xanthorrhoea gracilis*	2006	AuWe_VHS15860	-	
102	VHS15901	Australia	Australia	-	Forest soil	2006	AuWe_VHS15901	-	
103	VHS15910	Australia	Australia	WA, Warren SF	*Xanthorrhoea gracilis*	2006	AuWe_VHS15910	-	
104	VHS15913	Australia	Australia	WA, Lake Muir SF	Forest soil	2006	AuWe_VHS15913	-	
105	VHS16158	Australia	Australia	WA, Gnangara SF	*Banksia menziesii*	2006	AuWe_VHS16158	-	
106	VHS16171	Australia	Australia	WA, Quindanning	*Patersonia* spp.	2006	AuWe_VHS16171	-	
107	VHS16540	Australia	Australia	WA, Karnup	*Banksia littoralis*	2006	AuWe_VHS16540	-	
108	VHS16803	Australia	Australia	WA, Smith Brook	Forest soil	2007	AuWe_VHS16803	-	
109	VHS16810	Australia	Australia	WA, Smith Brook	*Patersonia* spp.	2007	AuWe_VHS16810	-	
110	VHS16850	Australia	Australia	WA, Gardner SF	*Patersonia* spp.	2007	AuWe_VHS16850	-	
111	VHS16904	Australia	Australia	WA, Yallingup	*Patersonia* spp.	2007	AuWe_VHS16904	-	
112	VHS17111	Australia	Australia	WA, Barlee Brook SF	*Patersonia* spp.	2007	AuWe_VHS1711	-	
113	VHS17241	Australia	Australia	WA, Wellard	*Stirlingia latifolia*	2007	AuWe_VHS17241	-	
114	VHS18950	Australia	Australia	-	*Dryandra formosa*	2007	AuWe_VHS18950	-	
115	VHS19381	Australia	Australia	WA, Warren SF	*Patersonia* spp.	2008	AuWe_VHS19381	-	
116	VHS19499	Australia	Australia	WA, Bannister	*Xanthorrhoea gracilis*	2008	AuWe_VHS19499	-	
117	VHS19556	Australia	Australia	WA, Lake Muir SF	*Patersonia longifolia*	2008	AuWe_VHS19556	-	
118	VHS19557	Australia	Australia	WA, Lake Muir SF	*Patersonia* spp.	2008	AuWe_VHS19557	-	
119	VHS19558	Australia	Australia	WA, Boorara SF	*Patersonia longifolia*	2008	AuWe_VHS19558	-	
120	VHS19562	Australia	Australia	WA, Mount Claremont	*Banksia* spp.	2008	AuWe_VHS19562	-	
121	VHS19629	Australia	Australia	WA, Warren SF	Forest soil	2008	AuWe_VHS19629	-	
122	VHS19905	Australia	Australia	WA, Jandakot	*Patersonia* spp.	2008	AuWe_VHS19905	-	
123	VHS21190	Australia	Australia	WA, Donnelly SF	*Patersonia* spp.	2009	AuWe_VHS21190	-	
124	VHS21191	Australia	Australia	WA, Donnelly SF	*Patersonia* spp.	2009	AuWe_VHS21191	-	
125	VHS21194	Australia	Australia	WA, Donnelly SF	*Patersonia* spp.	2009	AuWe_VHS21194	-	
126	VHS21247	Australia	Australia	WA, Warren SF	*Podocarpus drouynianus*	2009	AuWe_VHS21247	-	
127	VHS21882	Australia	Australia	WA, Binningup	*Banksia attenuata*	2009	AuWe_VHS21882	-	
128	VHS21883	Australia	Australia	WA, Binningup	*Banksia attenuata*	2009	AuWe_VHS21883	-	
129	VHS22022	Australia	Australia	WA, Nilgen NR	*Banksia attenuata*	2009	AuWe_VHS22022	-	
130	VHS22036	Australia	Australia	WA, Mimegarra	*Banksia attenuata*	2009	AuWe_VHS22036	-	
131	VHS22149	Australia	Australia	WA, Ellis Creek SF	*Patersonia* spp.	2009	AuWe_VHS22149	-	
132	VHS22151	Australia	Australia	WA, Ellis Creek SF	*Banksia grandis*	2009	AuWe_VHS22151	-	
133	VHS22154	Australia	Australia	WA, Ellis Creek SF	*Persoonia longifolia*	2009	AuWe_VHS22154	-	
134	VHS22157	Australia	Australia	WA, Nannup SF	*Persoonia longifolia*	2009	AuWe_VHS22157	-	
135	VHS22173	Australia	Australia	WA, Nannup SF	*Patersonia* spp.	2009	AuWe_VHS22173	-	
136	VHS22239	Australia	Australia	WA, Fitzgerald River NP	*Adenanthos velutinus*	2009	AuWe_VHS22239	-	
137	VHS22248	Australia	Australia	WA, Fitzgerald River NP	*Xanthorrhoea platyphylla*	2009	AuWe_VHS22248	-	
138	VHS22723	Australia	Australia	WA, Donnelly SF	*Persoonia longifolia*	2009	AuWe_VHS22723	-	
139	VHS22738	Australia	Australia	WA, Banyowla	*Hakea undulata*	2009	AuWe_VHS22738	-	
140	VHS22750	Australia	Australia	WA, Donnelly SF	*Patersonia* spp.	2009	AuWe_VHS22750	-	

141	VHS22760	Australia	Australia	WA, North Donnelly SF	*Xanthorrhoea gracilis*	2010	AuWe_VHS22760	-
142	VHS23189	Australia	Australia	WA, Dwellingup SF	*Bossiaea aquifolium*	2010	AuWe_VHS23189	-
143	VHS23193	Australia	Australia	WA, Dwellingup SF	*Bossiaea aquifolium*	2010	AuWe_VHS23193	-
144	VHS23294	Australia	Australia	WA, College Grove	*Banksia attenuata*	2010	AuWe_VHS23294	-
145	VHS23478	Australia	Australia	WA, Donnelly SF	*Patersonia* spp.	2010	AuWe_VHS23478	-
146	VHS23499	Australia	Australia	WA, Lake Muir SF	*Banksia* spp.	2010	AuWe_VHS23499	-
147	VHS23601	Australia	Australia	WA, Southampton	*Patersonia* spp.	2010	AuWe_VHS23601	-
148	VHS2386	Australia	Australia	WA, Lake Muir SF	*Podocarpus drouynianus*	1997	AuWe_VHS2386	-
149	VHS24254	Australia	Australia	WA, Dwellingup SF	*Bossiaea aquifolium*	2010	AuWe_VHS24254	-
150	VHS24275	Australia	Australia	WA, Nannup SF	*Podocarpus drouynianus*	2011	AuWe_VHS24275	-
151	VHS2431	Australia	Australia	WA, Warren SF	*Patersonia* spp.	1997	AuWe_VHS2431	-
152	VHS24384	Australia	Australia	WA, Cooljarloo	Xanthorrhoea drummondii	2010	AuWe_VHS24384	-
153	VHS24572	Australia	Australia	WA, Nannup SF	*Banksia grandis*	2011	AuWe_VHS24572	-
154	VHS24949	Australia	Australia	WA, Donnelly SF	*Patersonia* spp.	2011	AuWe_VHS24949	-
155	VHS25109	Australia	Australia	WA, O'Connor	*Leucopogon* spp.	2011	AuWe_VHS25109	-
156	VHS25131	Australia	Australia	WA, Ellis Creek SF	*Patersonia* spp.	2011	AuWe_VHS25131	-
157	VHS25143	Australia	Australia	WA, Warren SF	*Leucopogon verticillatus*	2011	AuWe_VHS25143	-
158	VHS25537	Australia	Australia	WA, Quinns Rock	Forest soil	2011	AuWe_VHS25537	-
159	VHS25594	Australia	Australia	WA, Fitzgerald River NP	*Banksia speciosa*	2011	AuWe_VHS25594	-
160	VHS25606	Australia	Australia	-	Forest soil	2011	AuWe_VHS25606	-
161	VHS25642	Australia	Australia	WA, Torndirrup NP	*Dryandra formosa*	2011	AuWe_VHS25642	-
162	VHS25826	Australia	Australia	WA, Dwellingup SF	*Bossiaea aquifolium*	2011	AuWe_VHS25826	-
163	VHS25842	Australia	Australia	WA, Dwellingup SF	*Eucalyptus* spp.	2011	AuWe_VHS25842	-
164	VHS25846	Australia	Australia	WA, Dwellingup SF	Forest soil	2011	AuWe_VHS25846	-
165	VHS25850	Australia	Australia	WA, Dwellingup SF	*Bossiaea aquifolium*	2011	AuWe_VHS25850	-
166	VHS25851	Australia	Australia	WA, Dwellingup SF	*Bossiaea aquifolium*	2011	AuWe_VHS25851	-
167	VHS25852	Australia	Australia	WA, Dwellingup SF	*Bossiaea aquifolium*	2011	AuWe_VHS25852	-
168	VHS25880	Australia	Australia	WA, Dwellingup SF	*Acacia* spp.	2011	AuWe_VHS25880	-
169	VHS26121	Australia	Australia	WA, Dwellingup SF	*Acacia* spp.	2011	AuWe_VHS26121	-
170	VHS26783	Australia	Australia	WA, Gosnells	*Mangifera indica*	2012	AuWe_VHS26783	-
171	VHS26910	Australia	Australia	WA, Fitzgerald River NP	*Banksia speciosa*	2012	AuWe_VHS26910	-
172	VHS26950	Australia	Australia	WA, Fitzgerald River NP	*Lambertia inermis*	2012	AuWe_VHS26950	-
173	VHS27353	Australia	Australia	WA, Nannup SF	*Astroloma* spp.	2012	AuWe_VHS27353	-
174	VHS27354	Australia	Australia	WA, Nannup SF	*Podocarpus drouynianus*	2012	AuWe_VHS27354	-
175	VHS27587	Australia	Australia	WA, Donnelly SF	*Patersonia umbrosa*	2012	AuWe_VHS27587	-
176	VHS27750	Australia	Australia	WA, North Donnelly SF	*Podocarpus drouynianus*	2012	AuWe_VHS27750	-
177	VHS27751	Australia	Australia	WA, North Donnelly SF	*Persoonia longifolia*	2012	AuWe_VHS27751	-
178	VHS2777	Australia	Australia	WA, North Donnelly SF	*Patersonia* spp.	1997	AuWe_VHS2777	-
179	VHS27966	Australia	Australia	WA, Witchcliffe	*Macrozamia riedlei*	2012	AuWe_VHS27966	-
180	VHS28069	Australia	Australia	WA, Yornup SF	*Patersonia* spp.	2012	AuWe_VHS28069	-
181	VHS28258	Australia	Australia	WA, Muchea	*Banksia menziesii*	2012	AuWe_VHS28258	-
182	VHS28281	Australia	Australia	WA, Margaret River	*Xanthorrhoea preissii*	2012	AuWe_VHS28281	-
183	VHS28588	Australia	Australia	WA, North Donnelly SF	*Banksia seminuda*	2013	AuWe_VHS28588	-
184	VHS28782	Australia	Australia	WA, William Bay NP	Forest soil	2013	AuWe_VHS28782	-
185	VHS28785	Australia	Australia	WA, William Bay NP	Forest soil	2013	AuWe_VHS28785	-
186	VHS2884	Australia	Australia	WA, Hilliger NP	*Patersonia* spp.	1997	AuWe_VHS2884	-
187	VHS29030	Australia	Australia	WA, Gardner SF	*Patersonia rudis*	2013	AuWe_VHS29030	-
188	VHS29031	Australia	Australia	WA, Gardner SF	*Patersonia rudis*	2013	AuWe_VHS29031	-
189	VHS29031R	Australia	Australia	WA, Gardner SF	*Patersonia rudis*	2013	AuWe_VHS29031R	-
190	VHS29032	Australia	Australia	WA, Gardner SF	*Lambertia formosa*	2013	AuWe_VHS29032	-
191	VHS29125	Australia	Australia	WA, Malaga	*Banksia attenuata*	2013	AuWe_VHS29125	-
192	VHS29331	Australia	Australia	WA, Redgate	*Patersonia occidentalis*	2013	AuWe_VHS29331	-
193	VHS3465	Australia	Australia	WA, Margaret River	Forest soil	2021	AuWe_VHS3465	-
194	VHS3972	Australia	Australia	WA, Nairn FB	*Leucopogon verticillatus*	2021	AuWe_VHS3972	-
195	VHS4521	Australia	Australia	WA, Greater Beedelup NP	*Persoonia longifolia*	1998	AuWe_VHS4521	-
196	VHS4564	Australia	Australia	WA, Collins	*Patersonia* spp.	1998	AuWe_VHS4564	-
197	VHS4595	Australia	Australia	WA, Warren SF	*Patersonia* spp.	1998	AuWe_VHS4595	-
198	VHS4632	Australia	Australia	WA, Blackwood SF	*Podocarpus drouynianus*	1998	AuWe_VHS4632	-
199	VHS4773	Australia	Australia	WA, Greater Beedelup NP	*Patersonia* spp.	1998	AuWe_VHS4773	-
200	VHS4828	Australia	Australia	WA, Donnelly SF	*Patersonia* spp.	1998	AuWe_VHS4828	-
201	VHS4975	Australia	Australia	WA, Nannup SF	*Patersonia* spp.	1998	AuWe_VHS4975	-
202	VHS4998	Australia	Australia	WA, Mount Frankland NP	*Patersonia* spp.	1998	AuWe_VHS4998	-
203	VHS5073	Australia	Australia	WA, Mount Frankland NP	*Patersonia* spp.	1998	AuWe_VHS5073	-
204	VHS6072	Australia	Australia	WA, William Bay NP	Forest soil	1999	AuWe_VHS6072	-
205	VHS6665	Australia	Australia	WA, Wilga SF	*Xanthorrhoea preissii*	1999	AuWe_VHS6665	-
206	VHS6682	Australia	Australia	WA, Mount Frankland NP	*Patersonia* spp.	1999	AuWe_VHS6682	-
207	VHS7057	Australia	Australia	WA, Warren SF	*Persoonia longifolia*	2000	AuWe_VHS7057	-
208	VHS7371	Australia	Australia	WA, Jarrahdale SF	*Patersonia* spp.	2000	AuWe_VHS7371	-
209	VHS7401	Australia	Australia	WA, Hilliger NP	*Banksia grandis*	1999	AuWe_VHS7401	-
210	VHS7411	Australia	Australia	WA, Wiltshire-Butler NP	*Patersonia* spp.	2000	AuWe_VHS7411	-
211	VHS7712	Australia	Australia	WA, Donnelly SF	*Patersonia* spp.	2000	AuWe_VHS7712	-
212	VHS7870	Australia	Australia	WA, Easter NP	*Xanthorrhoea gracilis*	2000	AuWe_VHS7870	-
213	VHS7971	Australia	Australia	WA, Easter NP	*Patersonia* spp.	2000	AuWe_VHS7971	-
214	VHS7981	Australia	Australia	WA, Easter NP	*Podocarpus drouynianus*	2000	AuWe_VHS7981	-
215	VHS8108	Australia	Australia	WA, Armadale	*Leucopogon spp.*	1998	AuWe_VHS8108	-
216	VHS8127	Australia	Australia	WA, Donnelly SF	*Patersonia* spp.	2000	AuWe_VHS8127	-

217	VHS8179	Australia	Australia	WA, Milyeannup SF	*Patersonia* spp.	2000	AuWe_VHS8179	-
218	VHS8320	Australia	Australia	WA, Middlesex	*Patersonia* spp.	2000	AuWe_VHS8320	-
219	VHS9672	Australia	Australia	WA, Donnelly SF	Forest soil	2001	AuWe_VHS9672	-
220	VHS9718	Australia	Australia	WA, Leeuwin-Naturaliste NP	*Banksia grandis*	2001	AuWe_VHS9718	-
221	VHS9778	Australia	Australia	WA, Donnelly SF	*Patersonia* spp.	2001	AuWe_VHS9778	-
222	SLPA109	Australia	Australia	VIC. Sugarloaf	Forest soil	2008	AuVi_SLPA109	EA01
223	SLPA109	Australia	Australia	VIC. Sugarloaf	Forest soil	2008	-	EA01_2
224	HAS2297	Australia	Australia	WA, Cooljarloo	*Banksia* spp.	1996	AuWe_HAS2297	HSA2297
225	VHS20916	Australia	Australia	WA, Hope Valley	*Banksia attenuata*	2009	AuWe_VHS20916	VHS20916
226	VHS20987	Australia	Australia	WA, North Donnelly SF	*Persoonia longifolia*	2009	AuWe_VHS20987	VHS20987
227	VHS21205	Australia	Australia	Leeuwin-Naturaliste NP	*Podocarpus drouynianus*	2009	AuWe_VHS21205	VHS21205
228	VHS21474	Australia	Australia	WA, Wellington NP	*Banksia grandis*	2009	AuWe_VHS21474	VHS21474
229	VHS21692	Australia	Australia	WA, Tamala Park	*Banksia attenuata*	2009	AuWe_VHS21692	VHS21692
230	VHS21881	Australia	Australia	WA, Binningup	*Banksia attenuata*	2009	AuWe_VHS21881	VHS21881
231	VHS22472	Australia	Australia	WA, Nilgen NR	*Banksia attenuata*	2009	AuWe_VHS22472	VHS22472
232	VHS22575	Australia	Australia	WA, Cooljarloo	*Banksia menziesii*	2009	-	VHS22575
233	VHS22754	Australia	Australia	WA, North Donnelly SF	*Persoonia longifolia*	2009	AuWe_VHS22754	VHS22754
234	VHS8081	Australia	Australia	WA, Greater Hawke NP	*Patersonia* spp.	2000	AuWe_VHS8081	VHS8081
235	WAC13202	Australia	Australia	WA, Yalgorup	*Eucalyptus gomphocephala*	2008	AuWe_WAC13202	WAC13202
236	WAC13203	Australia	Australia	WA, Yalgorup	*Agonis flexuosa*	2008	AuWe_WAC13203	WAC13203
237	WAC13205	Australia	Australia	WA, Jarrahdale	*Eucalyptus marginata*	2008	AuWe_WAC13205	WAC13205
238	WAC13200	Australia	Australia	WA, Yalgorup	*Eucalyptus gomphocephala*	2008	AuWe_WAC13200	WAC13200
239	WAC13201	Australia	Australia	WA, Yalgorup	*Eucalyptus marginata*	2008	AuWe_WAC13201	WAC13201
240	WAC13201	Australia	Australia	WA, Yalgorup	*Eucalyptus marginata*	2008	-	WAC13201-1
241	WAC13201	Australia	Australia	WA, Yalgorup	*Eucalyptus marginata*	2008	-	WAC13201-2
242	WAC13204	Australia	Australia	WA, Yalgorup	*Eucalyptus gomphocephala*	2008	AuWe_WAC13204	WAC13204
243	DDS3700	Australia	Australia	WA, Sutton FB	Forest soil	1995	AuWe_DDS3700	DDS3700
244	W1838	Australia	Australia	NSW, Wollemi National Park	*Wollemia nobilis*	2009	AuWa_W1838	EA03
245	W1839	Australia	Australia	NSW, Wollemi National Park	*Wollemia nobilis*	2009	AuWa_W1839	EA04
246	W1839	Australia	Australia	NSW, Wollemi National Park	*Wollemia nobilis*	2009	-	EA04_6
247	HAS1153	Australia	Australia	WA, Eneabba	*Banksia* spp.	1991	AuWe_HAS1153	HSA1153
248	VHS15041	Australia	Australia	WA, Banksiadale SF	*Eucalyptus marginata*	2005	-	VHS15041
249	VHS15316	Australia	Australia	WA, Donnelly SF	*Podocarpus drouynianus*	2006	AuWe_VHS15316	VHS15316
250	VHS16260	Australia	Australia	WA, Gnangara SF	*Banksia attenuata*	2006	AuWe_VHS16260	VHS16260
251	VHS21011	Australia	Australia	WA, Cape Arid NP	*Xanthorrhoea preissii*	2009	AuWe_VHS21011	VHS21011
252	VHS21076	Australia	Australia	WA, Yelverton	*Podocarpus drouynianus*	2009	AuWe_VHS21076	VHS21076
253	VHS21416	Australia	Australia	WA, Fitzgerald River NP	*Banksia media*	2009	AuWe_VHS21416	VHS21416
254	VHS22131	Australia	Australia	WA, Gracetown	*Xanthorrhoea preissii*	2009	AuWe_VHS22131	VHS22131
255	VHS22240	Australia	Australia	WA, Fitzgerald River NP	*Adenanthos velutinus*	2009	AuWe_VHS22240	VHS22240
256	VHS22497	Australia	Australia	WA, Malaga	*Banksia menziesii*	2009	AuWe_VHS22497	VHS22497
257	VHS22509	Australia	Australia	WA, Nannup SF	*Patersonia* spp.	2009	AuWe_VHS22509	VHS22509
258	VHS22528	Australia	Australia	WA, Nannup SF	*Patersonia* spp.	2009	AuWe_VHS22528	VHS22528
259	CI2	Canary Islands	Spain	-	Forest soil	2012	Ca_CI2	-
260	CI4	Canary Islands	Spain	-	Forest soil	2012	Ca_CI4	-
261	CI8	Canary Islands	Spain	-	Forest soil	2012	Ca_CI8	-
262	CI10	Canary Islands	Spain	-	Forest soil	2012	Ca_CI10	-
263	CI13	Canary Islands	Spain	-	Forest soil	2012	Ca_CI13	-
264	CI15	Canary Islands	Spain	-	Forest soil	2012	Ca_CI15	-
265	CI16	Canary Islands	Spain	-	Forest soil	2012	Ca_CI16	-
266	CI17	Canary Islands	Spain	-	Forest soil	2012	Ca_CI17	-
267	CI19	Canary Islands	Spain	-	Forest soil	2012	Ca_CI19	-
268	CI21	Canary Islands	Spain	-	Forest soil	2012	Ca_CI21	-
269	CI24	Canary Islands	Spain	-	Forest soil	2012	Ca_CI24	-
270	CI27	Canary Islands	Spain	-	Forest soil	2012	Ca_CI27	-
271	CI28	Canary Islands	Spain	-	Forest soil	2012	Ca_CI28	-
272	CI29	Canary Islands	Spain	-	Forest soil	2012	Ca_CI29	-
273	CI31	Canary Islands	Spain	-	Forest soil	2012	Ca_CI31	-
274	CI32	Canary Islands	Spain	-	Forest soil	2012	Ca_CI32	-
275	CI33	Canary Islands	Spain	-	Forest soil	2012	Ca_CI33	-
276	CI35	Canary Islands	Spain	-	Forest soil	2012	Ca_CI35	-
277	CI36	Canary Islands	Spain	-	Forest soil	2012	Ca_CI36	-
278	CI1	Canary Islands	Spain	-	Forest soil	2012	Ca_CI1	CAN01
279	CI3	Canary Islands	Spain	-	Forest soil	2012	Ca_CI3	CAN03
280	CI5	Canary Islands	Spain	-	Forest soil	2012	Ca_CI5	CAN05
281	CI6	Canary Islands	Spain	-	Forest soil	2012	Ca_CI6	CAN06
282	CI7	Canary Islands	Spain	-	Forest soil	2012	Ca_CI7	CAN07
283	CI9	Canary Islands	Spain	-	Forest soil	2012	Ca_CI9	CAN09
284	CI11	Canary Islands	Spain	-	Forest soil	2012	Ca_CI11	CAN11
328	BEX2H	North America	United States	OR	Forest soil	2000-2012	US_BEX2H	US01
329	BEX3	North America	United States	OR	Forest soil	2000-2012	US_BEX3	US02
312	CBS113347	New Zealand	New Zealand	-	Forest soil	2000-2012	NW_CBS113347	CBS113347
313	CBS119108	New Zealand	New Zealand	-	Forest soil	2000-2012	NW_CBS119108	CBS119108
295	CS102	Europe	Switzerland	-	*Rhododendron Azzuro*	2010	EuSw_CS102	EU02
296	CS102	Europe	Switzerland	-	*Rhododendron Azzuro*	2010	-	EU02-1
297	CS102	Europe	Switzerland	-	*Rhododendron Azzuro*	2010	-	EU02-2
327	CS266	North America	United States	OR, Multnomah, Gresham	Water	2010	US_CS266	-

332	CS267	North America	United States	OR, Multnomah, Gresham	*Rhododendron* spp.	2010	US_CS267	US05
333	CS268	North America	United States	OR, Multnomah, Gresham	*Rhododendron* spp.	2010	-	US06
334	CS269	North America	United States	OR, Multnomah, Gresham	*Rhododendron* spp.	2010	US_CS269	US07
335	CS270	North America	United States	OR, Multnomah, Gresham	*Rhododendron* spp.	2010	-	US08
285	CS310	Europe	Belgium	-	*Rhododendron* spp.	2006	EuBe_CS310	-
288	CS315	Europe	Belgium	-	*Rhododendron* spp.	2009	EuBe_CS315	EU03
289	CS483	Europe	Czech Republic	Moravia	*Rhododendron catawbiense Grandiflorum*	2000-2012	-	EU05
286	CS486	Europe	Czech Republic	Moravia	*Alnus glutinosa*	2000-2012	EuCz_CS486	-
290	CS488	Europe	Czech Republic	Moravia	*Quercus robur*	2000-2012	-	EU06
291	CS501	Europe	France	Corsica	*Antirrhinum majus*	2000-2012	-	EU07
330	EE9488517	North America	United States	-	Forest soil	2000-2012	US_EE9488517	US03
331	EE9488531	North America	United States	-	Forest soil	2000-2012	US_EE9488531	US04
314	NZ1072	New Zealand	New Zealand	WN, Bolton Street Cemeter	*Elaeocarpus dentatus*	2000-2012	NW_NZ1072	NZ1072
315	NZ11702	New Zealand	New Zealand	GB, Ormsby	*Pinus radiata*	2000-2012	NW_NZ11702	NZ011702
304	NZ11703	New Zealand	New Zealand	-	*Cytisus scoparius*	2000-2012	NW_NZ11703	-
305	NZ117A	New Zealand	New Zealand	TO, Rotoaira Forest	*Malus* spp.	2000-2012	NW_NZ117A	-
316	NZ2750	New Zealand	New Zealand	AK, Auckland, Kumeu, Riverhead	Forest soil	2000-2012	NW_NZ2750	NZ2750
317	NZ2751	New Zealand	New Zealand	AK, Auckland	*Araucaria heterophylla*	2000-2012	NW_NZ2751	NZ2751
306	NZ2815	New Zealand	New Zealand	AK, Northshore	*Araucaria heterophylla*	2000-2012	NW_NZ2815	-
307	NZ2816	New Zealand	New Zealand	AK, Cornwall Park	*Araucaria heterophylla*	2000-2012	NW_NZ2816	-
308	NZ3061	New Zealand	New Zealand	BP, Kinleith Forest	Forest soil	2000-2012	NW_NZ3061	-
309	NZ3070	New Zealand	New Zealand	BP, Kinleith Forest	Forest soil	2000-2012	NW_NZ3070	-
318	NZ3376	New Zealand	New Zealand	WO, Hamilton	Forest soil	2000-2012	NW_NZ3376	NZ3376
319	NZ3377	New Zealand	New Zealand	AK, Southdown	*Vitex lucens*	2000-2012	NW_NZ3377	NZ3377
320	NZ3378	New Zealand	New Zealand	AK, Penrose	*Idesia polycarpa*	2000-2012	NW_NZ3378	NZ3378
321	NZ3448	New Zealand	New Zealand	AK, Auckland	*Metrosideros kermadecensis*	2000-2012	NW_NZ3448	NZ3448
322	NZ3448	New Zealand	New Zealand	AK, Auckland	*Metrosideros kermadecensis*	2000-2012	-	NZ3448-2
323	NZ3448	New Zealand	New Zealand	AK, Auckland	*Metrosideros kermadecensis*	2000-2012	-	NZ3448-3
324	NZ3448	New Zealand	New Zealand	AK, Auckland	*Metrosideros kermadecensis*	2000-2012	-	NZ3448-4
325	NZ3600	New Zealand	New Zealand	AK, Auckland, Albert Park	*Pinus radiata*	2000-2012	NW_NZ3600	NZ3600
310	NZ571	New Zealand	New Zealand	NN, Nelson	*Cordyline australis*	2000-2012	NW_NZ571	-
326	NZ951	New Zealand	New Zealand	AK, Onehunga, Waikaraka Cemetery	*Pittosporum crassifolium*	2000-2012	NW_NZ951	NZ0951
311	NZ965	New Zealand	New Zealand	ND, Onepu	*Pinus radiata*	2000-2012	NW_NZ965	-
292	PG217	Europe	Spain	-	*Genista* spp.	2007	EuSp_PG217	EU08
298	PG270	Europe	Spain	-	Forest soil	2000-2012	EuSp_PG270	EU09
299	PG703	Europe	Spain	-	Forest soil	2000-2012	EuSp_PG703	EU10
293	PG827	Europe	Spain	-	*Pittosporum* spp.	2009	EuSp_PG827	EU11
287	TAVIRA1	Europe	Portugal	-	Forest soil	2000-2012	EuPo_TAVIRA	-
294	TAVIRA2	Europe	Portugal	-	Forest soil	2000-2012	-	EU01
300	UK104109	Europe	United Kingdom	Surrey, RHS Gardens	*Lyonothamnus* spp.	2000-2012	UK_UK104109	-
301	UK226	Europe	United Kingdom	Surrey, RHS Gardens	*Hebe* spp.	2000-2012	UK_UK226	EU13
302	UK46329	Europe	United Kingdom	Surrey, RHS Gardens	*Lyonothamnus* spp.	2012	UK_UK46329	EU14
303	UK46329	Europe	United Kingdom	Surrey, RHS Gardens	*Lyonothamnus* spp.	2012	-	EU14_2

Figure S1. Deviation from Hardy-Weinberg equilibrium in 10 SSR loci in six subpopulation.

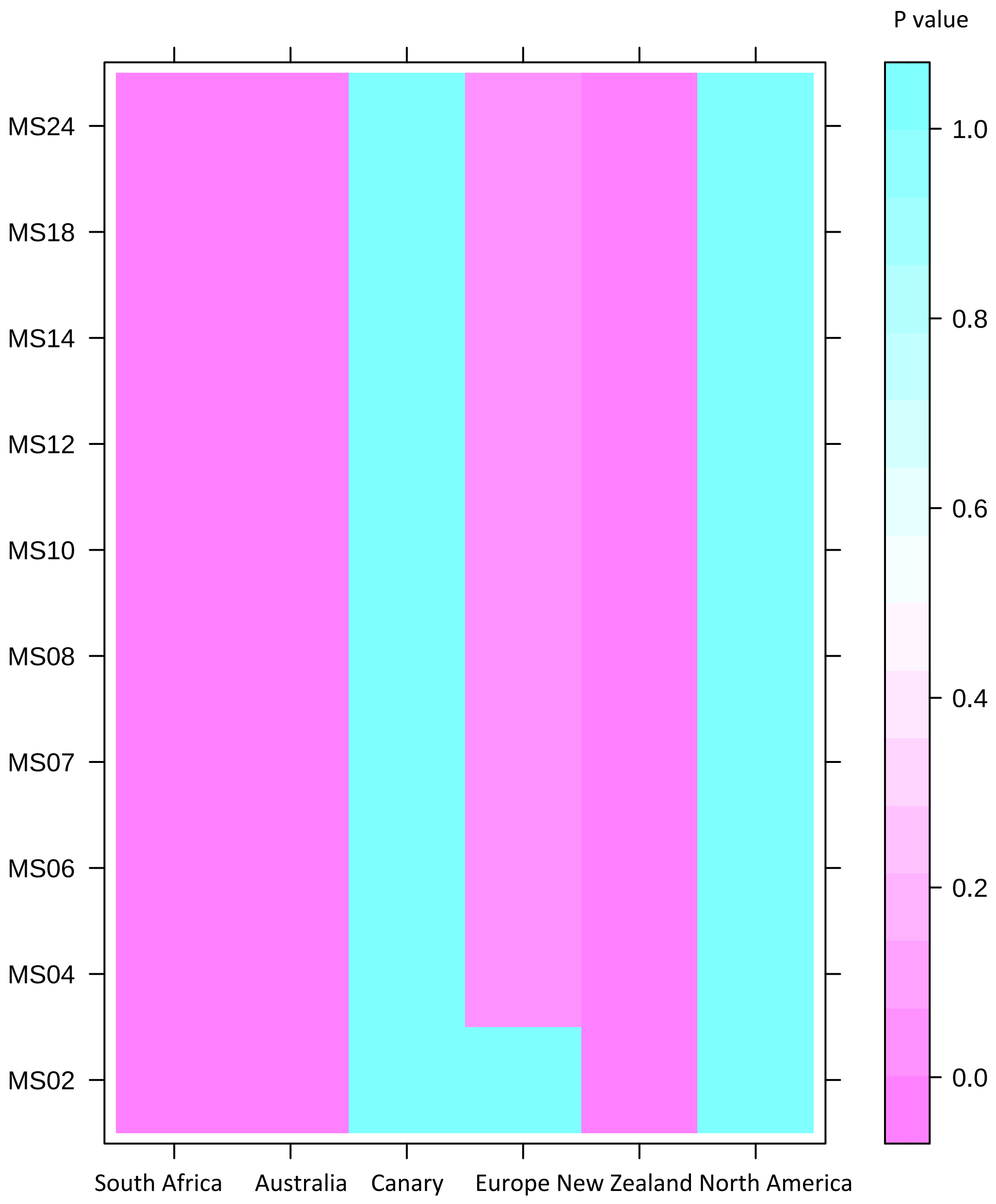

Figure S2. Pair-wise linkage disequilibrium among 10 SSR loci and in six subpopulations

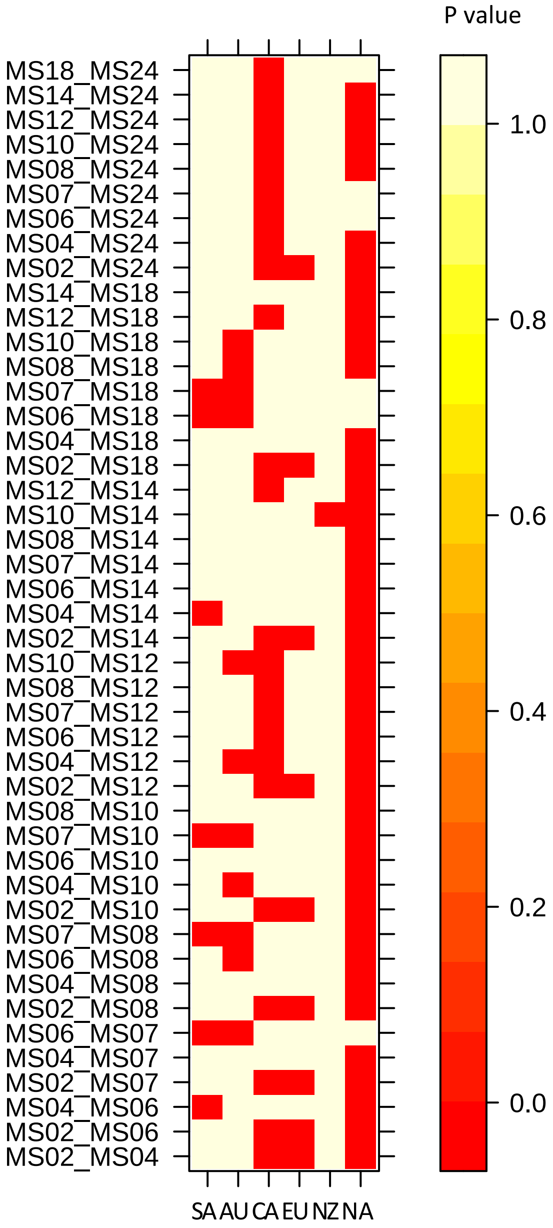

Figure S3. STRUCTURE results using 10 SSR loci in six geographic populations of *Phytophthora multivora*.Barplots representing the average estimated membership probability (y-axis) of an individual assigned to a specific cluster (indicated by specific color) among number of cluster computed. The cluster numbers with four highest ΔK marked brown in the right from each barplots.

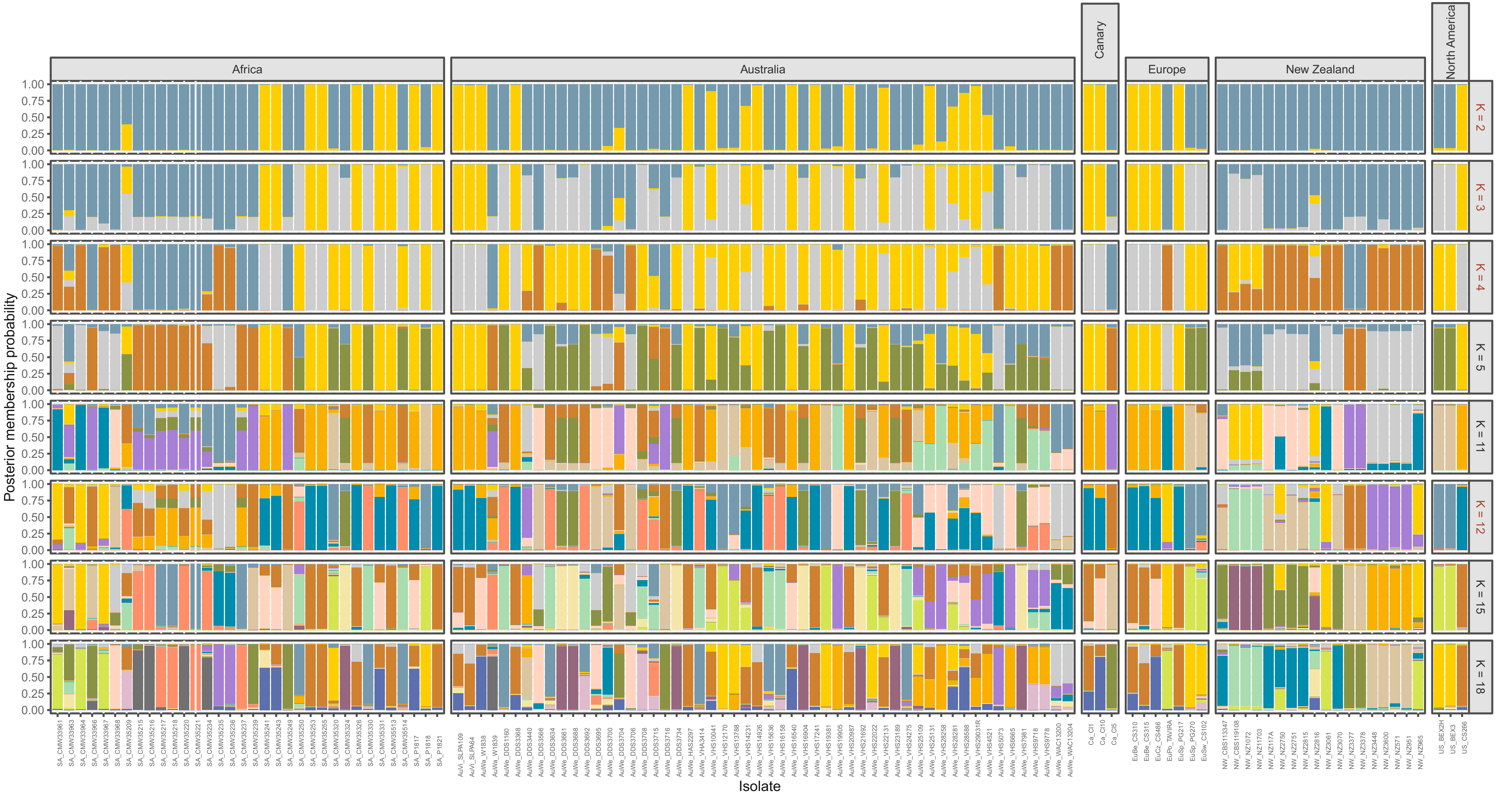

Notes 1. Details on the Approximate Bayesian Computational analysis

Six different scenarios of the demographic history for three subpopulations graphically described in Figure S4. In a first step in order to define likelihood scenario of demographic events in three the most genetically diverse subpopulations, i.e. South African, Australian and New Zealand we simulated 6x10^6 pseudo-observed datasets (PODs) under uniform distribution of the parameters in a broad ranges: 2-1000 of effective population sizes for the initially introduced subpopulations (NZb, AUb or Sab) and 10-10000 for the sampled in t0 subpopulations, with conditions that establishing (indicated with “b”) subpopulations smaller by effective population size than a source and resulted subpopulation, e.g. AUb<AU, AUb<SA, and t2 > t1 in range 10-10000 generations and db lag of establishing subpopulation from 1-10000. In a second step we conducted compuations for all six geographic subpopulations considering results of the first step for assuming likelihood competing scenario as graphically described in Figure S6. We used identical parameters and conditions in the first step for simulating PODs and in addition we set range of admixture r1 from 0.001 to 0.999. In both steps, for each simulation, a value for each parameter was drawn from prior distribution and performed coalescent simulations with the same number of alleles and loci per population as in the observed dataset. A set of 15 summary statistics describing within and among population genetic diversity were calculated for each POD and the observed data. Population specific statistics included mean number of alleles in population (NAL); allele size variance (VAR). Between population statistics included FST , mean variance of the absolute allelic size (V2P) and mean number of alleles (NAL). We used for simulation generalized stepwise-mutation model and default parameters of the DIYABC v.2.1.0.

The posterior probabilities of each competing scenario were estimated using a logistic regression on the 1000 of closest to the observed dataset of 6x10^6 simulated datasets. The best-fitting scenario was selected based on the highest posterior probability with a non-overlapping 95% confidence interval. We evaluated the ability of the ABC analysis to discriminate between the competing scenarios by analyzing subset of 500 of closest to observed simulated data sets. Therefore, we estimated the Type-I error rate as the proportion of occurrences the best-supported scenario did not show the highest posterior probability among the competing scenarios and we estimated the Type-II error rate, by calculating the mean proportion of occurrences in which the best-supported model was incorrectly supported instead of selected for simulation one of the competing scenario.

For the best supported scenario the posterior probability distribution of time and demographic parameters (see Table S3) were estimated, after a logit transformation, by local linear regression on the 1% of simulations closest to the observed data. Finally, for model checking we simulated 10000 PODs from the posterior under the best supported scenario in order to evaluate whether this model could successfully reproduce the observed data.

Figure S4. Six scenario of the demographic events assumed for the first step of ABC evaluation. Time is not scaled on the schemes. Supported scenario marked with orange rectangle.
Sampled subpopulations: NZ - New Zealand, SA - South Africa, AU - Australia; those abbreviations with additional "b"- introduced and establishing subpopulations with limited effective population sizes; t - nominal time of demographic event; t-db- nominal time of establishing.

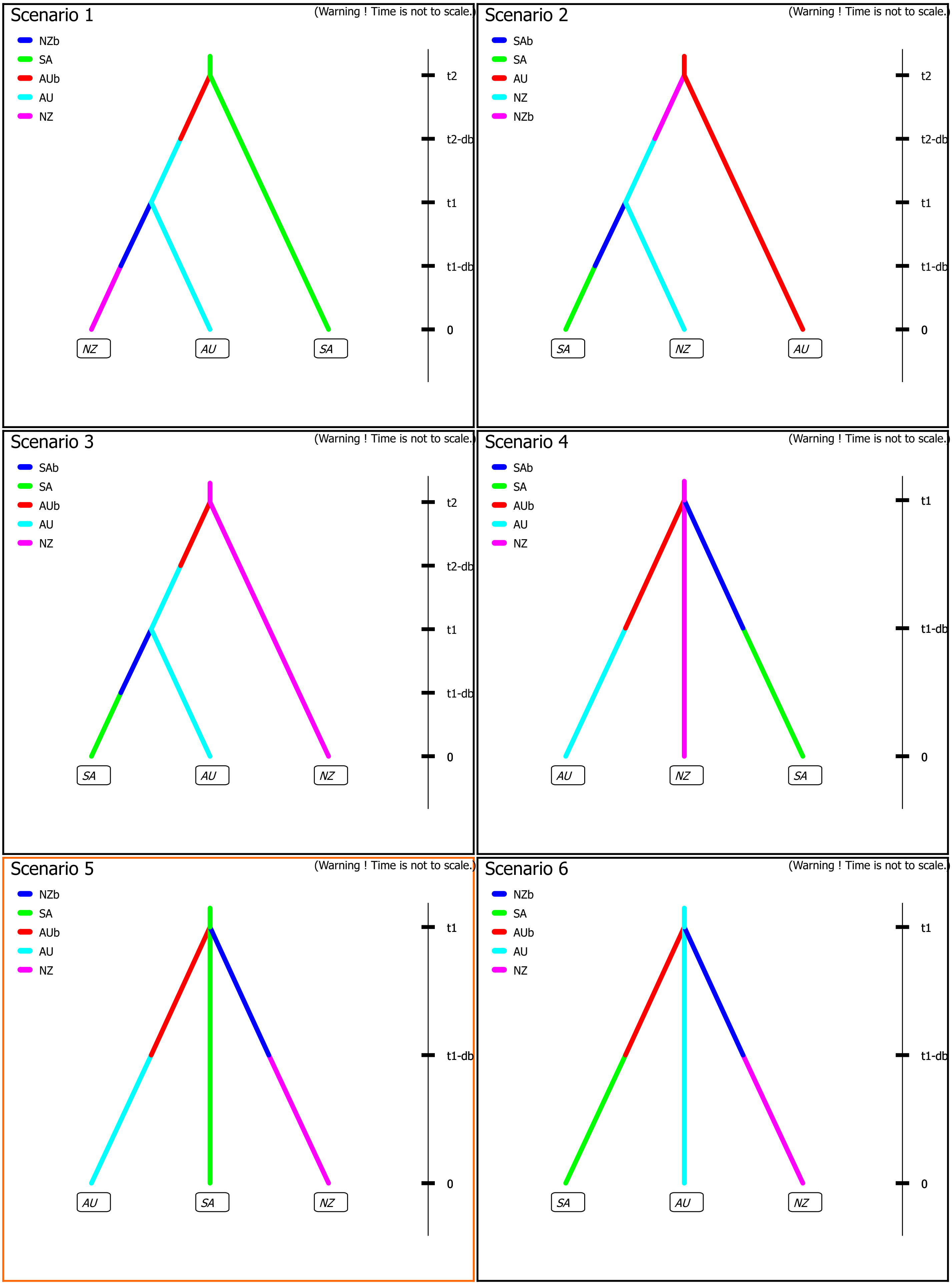

Figure S5. Figure S5. Mean posterior probabilities of the competing models for ABC analysis of the first step.

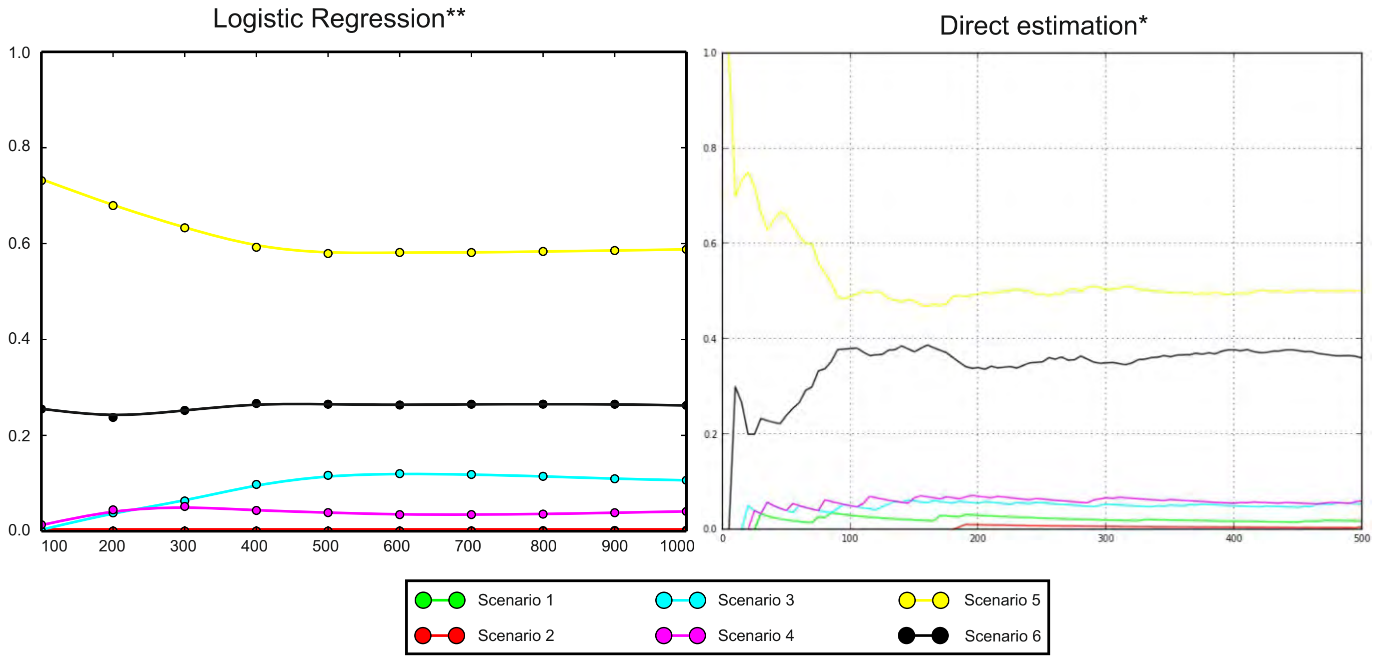

* Posterior probabilities inferred directly from summary statistics of the 500 closest to observed of $6x10^6$ simulated datasets;

** Posterior probabilities inferred from linear discriminants of the summary statistics inferred from 1000 closest to observed of $6x10^6$ simulated datasets.

Figure S6. Six scenario of the demographic events assumed for the second step of ABC evaluation. Time is not scaled on the schemes. Supported scenario marked with orange rectangle.
Sampled subpopulations: NZ - New Zealand, SA - South Africa, AU - Australia; CA- Canary Islands; US - North America; EU - Europe; those abbreviations with additional "b"- introduced and establishing subpopulations with limited effective population sizes; t - nominal time of demographic event; t-db- nominal time of establishing.

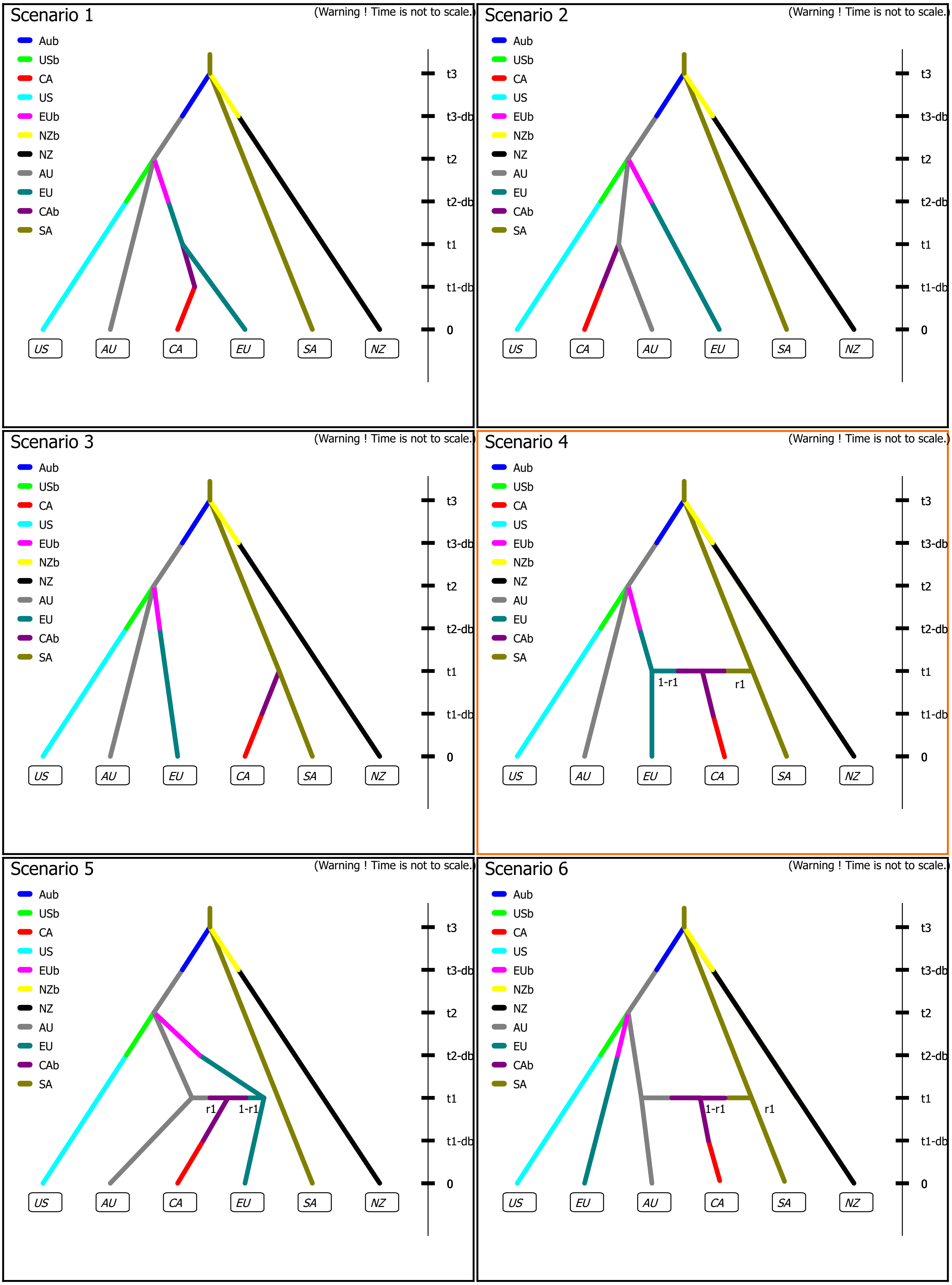

Figure S7. Mean posterior probabilities of the competing models for ABC analysis of the second step.

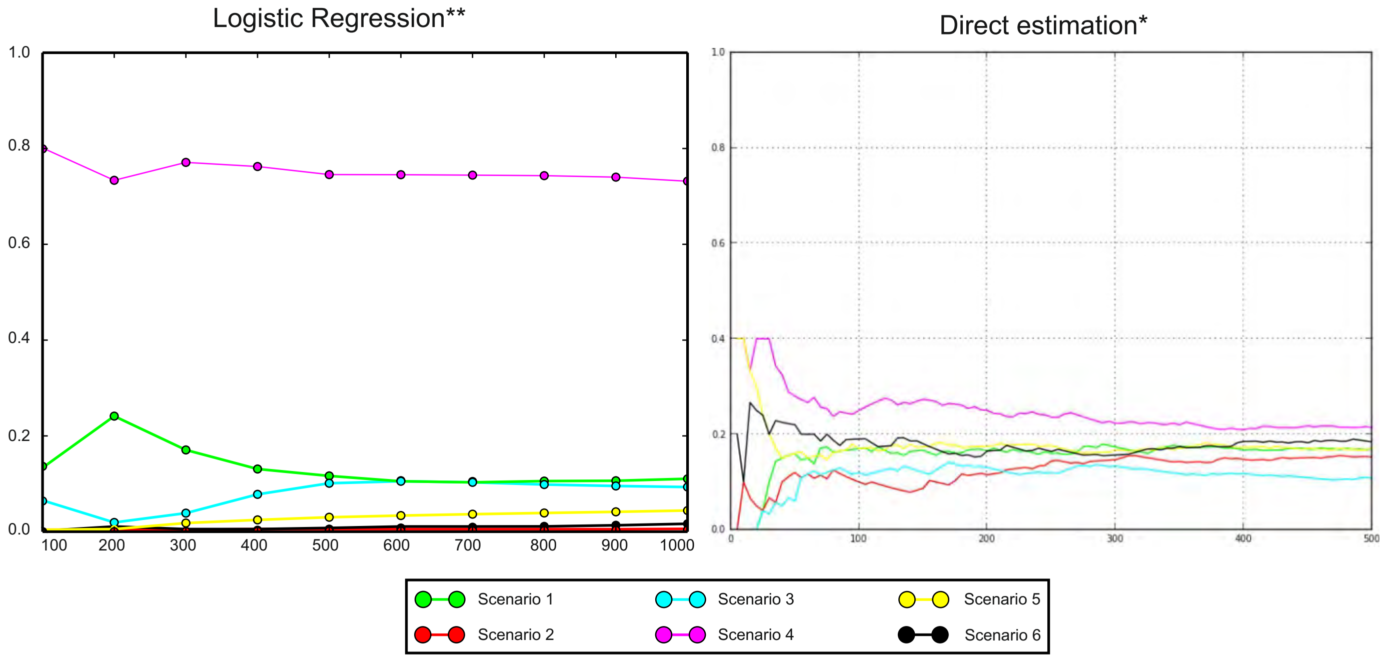

* Posterior probabilities inferred directly from summary statistics of the 500 closest to observed of 6x10^6 simulated data sets;

** Posterior probabilities inferred from linear discriminants of the summary statistics inferred from 1000 closest to observed of 10^6 simulated datasets.

Table S2. Mean posterior probabilities of the competing models for ABC analysis inferred from the closest to observed of $6x10^6$ simulated datasets.

Post. prob. 0.95 CI	S1	S2	S3	S4	S5	S6	I type error	II type error
First step								
*Direct	0.02±0.01	0.01±0.00	0.05±0.01	0.06±0.01	0.51±0.05	0.35±0.04	0.20	0.06
**LDA	0.00±0.00	0.00±0.00	0.09±0.04	0.04±0.01	0.61±0.05	0.26±0.01	0.21	0.05
Second step								
*Direct	0.17±0.01	0.13±0.02	0.11±0.02	0.24±0.02	0.17±0	0.18±0.02	0.40	0.06
**LDA	0.13±0.04	0.00±0.00	0.08±0.03	0.75±0.02	0.03±0.02	0.01±0.01	0.16	0.04

Table S3. Model checking of the likelihood scenario for ABC analysis

**First step**			**Second step**		
Summary stat.	**Obs. value**	**Simulated**	**Summary stat.**	**Obs. value**	**Simulated**
HET_1_1	0.6006	0.875	HET_1_1	0.60	0.63
HET_1_2	0.5574	0.7905	HET_1_2	0.56	0.56
HET_1_3	0.513	0.541	HET_1_3	0.37	0.11
MGW_1_1	0.7385	0.139	HET_1_4	0.43	0.26
MGW_1_2	0.7328	0.12	HET_1_5	0.51	0.47
MGW_1_3	0.7872	0.3075	HET_1_6	0.29	0.05
H2P_1_1&2	0.5895	0.765	MGW_1_1	0.74	0.11
H2P_1_1&3	0.6148	0.816	MGW_1_2	0.73	0.11
H2P_1_2&3	0.5814	0.7645	MGW_1_3	0.81	0.81
LIK_1_1&2	1.24	0.504	MGW_1_4	0.91	0.94
LIK_1_1&3	1.472	0.8165	MGW_1_5	0.79	0.40
LIK_1_2&1	1.0828	0.401	MGW_1_6	0.73	0.55
LIK_1_2&3	1.3821	0.752	H2P_1_1&2	0.59	0.53
LIK_1_3&1	1.3182	0.638	H2P_1_1&3	0.59	0.56
LIK_1_3&2	1.4672	0.6885	H2P_1_1&4	0.59	0.52
DAS_1_1&2	0.3883	0.36	H2P_1_1&5	0.61	0.62
DAS_1_1&3	0.3453	0.2215	H2P_1_1&6	0.60	0.57
DAS_1_2&3	0.3718	0.306	H2P_1_2&3	0.56	0.54
DM2_1_1&2	0.1571	0.126	H2P_1_2&4	0.55	0.51
DM2_1_1&3	0.3059	0.312	H2P_1_2&5	0.58	0.57
DM2_1_2&3	0.3019	0.2775	H2P_1_2&6	0.55	0.52
-	-	-	H2P_1_3&4	0.43	0.21
-	-	-	H2P_1_3&5	0.55	0.54
-	-	-	H2P_1_3&6	0.43	0.13
-	-	-	H2P_1_4&5	0.56	0.49
-	-	-	H2P_1_4&6	0.40	0.11
-	-	-	H2P_1_5&6	0.54	0.48
-	-	-	LIK_1_1&2	1.24	0.47
-	-	-	LIK_1_1&3	1.13	0.35
-	-	-	LIK_1_1&4	1.29	0.48
-	-	-	LIK_1_1&5	1.47	0.65

-	-	-	LIK_1_1&6	1.28	0.47
-	-	-	LIK_1_2&1	1.08	0.40
-	-	-	LIK_1_2&3	1.17	0.53
-	-	-	LIK_1_2&4	1.04	0.45
-	-	-	LIK_1_2&5	1.38	0.57
-	-	-	LIK_1_2&6	1.03	0.40
-	-	-	LIK_1_3&1	0.85	0.18
-	-	-	LIK_1_3&2	0.88	0.28
-	-	-	LIK_1_3&4	0.84	0.32
-	-	-	LIK_1_3&5	1.59	0.70
-	-	-	LIK_1_3&6	1.11	0.45
-	-	-	LIK_1_4&1	0.98	0.26
-	-	-	LIK_1_4&2	0.85	0.33
-	-	-	LIK_1_4&3	0.88	0.32
-	-	-	LIK_1_4&5	1.43	0.61
-	-	-	LIK_1_4&6	0.76	0.10
-	-	-	LIK_1_5&1	1.32	0.67
-	-	-	LIK_1_5&2	1.47	0.67
-	-	-	LIK_1_5&3	1.57	0.76
-	-	-	LIK_1_5&4	1.31	0.50
-	-	-	LIK_1_5&6	1.42	0.64
-	-	-	LIK_1_6&1	0.98	0.29
-	-	-	LIK_1_6&2	0.73	0.19
-	-	-	LIK_1_6&3	1.12	0.47
-	-	-	LIK_1_6&4	0.58	0.05
-	-	-	LIK_1_6&5	1.37	0.56
-	-	-	DAS_1_1&2	0.39	0.53
-	-	-	DAS_1_1&3	0.45	0.71
-	-	-	DAS_1_1&4	0.41	0.62
-	-	-	DAS_1_1&5	0.35	0.36
-	-	-	DAS_1_1&6	0.41	0.64
-	-	-	DAS_1_2&3	0.45	0.64
-	-	-	DAS_1_2&4	0.48	0.69
-	-	-	DAS_1_2&5	0.37	0.46
-	-	-	DAS_1_2&6	0.51	0.79
-	-	-	DAS_1_3&4	0.56	0.82
-	-	-	DAS_1_3&5	0.31	0.27
-	-	-	DAS_1_3&6	0.49	0.75
-	-	-	DAS_1_4&5	0.36	0.43
-	-	-	DAS_1_4&6	0.61	0.93
-	-	-	DAS_1_5&6	0.37	0.44
-	-	-	DM2_1_1&2	0.16	0.14
-	-	-	DM2_1_1&3	0.54	0.36
-	-	-	DM2_1_1&4	0.42	0.30
-	-	-	DM2_1_1&5	0.31	0.27
-	-	-	DM2_1_1&6	0.53	0.29
-	-	-	DM2_1_2&3	0.51	0.42
-	-	-	DM2_1_2&4	0.27	0.37
-	-	-	DM2_1_2&5	0.30	0.24
-	-	-	DM2_1_2&6	0.29	0.28

-	-	-	DM2_1_3&4	0.18	0.12
-	-	-	DM2_1_3&5	0.84	0.43
-	-	-	DM2_1_3&6	0.58	0.30
-	-	-	DM2_1_4&5	0.72	0.40
-	-	-	DM2_1_4&6	0.18	0.08
-	-	-	DM2_1_5&6	0.76	0.37

*, **, *** - corresponding to significant to tail-area probabilities (p-values) < 0.05, < 0.01 and <0.001 respectively. Abbreviations for the summary statistics are as follows: mean number of alleles in subpopulation (NAL); mean expected heterozygosity in population (HET); mean size variance (VAR); mean Garcia-Williamson index (MGW); mean number of alleles pooling samples from subpopulations (N2P); mean expected heterozygosity pooling samples from populations (H2P); mean variance of the absolute allelic size pooling samples from populations (V2P); F_{ST} between 2 samples (FST); mean individual assignment likelihoods of population i assigned to population j (LIK); shared allele distance between populations (DAS); dμ2 distance (DM2).

Table S4. Parameter estimation for the likelihood scenario of the second step in natural units

Parameters	mean	median	mode	Q 2.5	Q 5.0	Q 25	Q 75	Q 95	Q 97.5
SA	3750.00	3520.00	3520.00	845.00	1160.00	2370.00	4730.00	7320.00	8460.00
AU	6200.00	6270.00	6580.00	2420.00	2760.00	4790.00	7700.00	9440.00	9630.00
CA	5430.00	5480.00	7870.00	901.00	1360.00	3180.00	7640.00	9540.00	9740.00
EU	4070.00	3450.00	2810.00	792.00	1010.00	2110.00	5750.00	8820.00	9300.00
NZ	5430.00	5260.00	3860.00	1730.00	2210.00	3800.00	6990.00	9190.00	9540.00
US	6320.00	6640.00	8620.00	1570.00	2120.00	4480.00	8350.00	9660.00	9840.00
t1	124.00	73.70	24.90	14.60	17.00	36.00	143.00	385.00	517.00
db	247.00	91.30	15.70	6.06	11.70	42.10	215.00	841.00	1390.00
CAb	548.00	565.00	577.00	74.10	131.00	339.00	756.00	938.00	971.00
r1	0.26	0.21	0.17	0.02	0.04	0.11	0.36	0.70	0.82
t2	392.00	271.00	86.10	47.20	58.10	146.00	492.00	1060.00	1340.00
USb	782.00	851.00	989.00	176.00	296.00	686.00	948.00	992.00	996.00
EUb	673.00	734.00	978.00	124.00	182.00	497.00	884.00	982.00	991.00
t3	854.00	450.00	184.00	75.40	101.00	234.00	868.00	3140.00	4580.00
Aub	594.00	625.00	875.00	54.00	105.00	374.00	843.00	972.00	990.00
NZb	487.00	467.00	171.00	43.70	75.10	244.00	714.00	930.00	966.00

* - effective population size is provided in term of diploid individuals; ** - time of the event are provided in generation before present. Qx: x % quantile.

Table S5. Statistical results of the MASCOT analysis

Summary Statistic	mean	stdErr of mean	stdev	variance	median	value range	geometric mean	95% HPD interval	auto-correlation time	effective sample	number of samples
posterior	-5657.595	0.2766	10.4727	109.6779	-5657.089	[-5701.0725; -5613.5212]	n/a	[-5679.0065; -5638.3373]	12562.3774	1433	9001
likelihood	-5678.789	0.3316	7.889	62.236	-5678.477	[-5716.7087; -5653.6365]	n/a	[-5694.1748; -5663.462]	31811.8464	565.9	9001
prior	21.1941	0.4103	9.4267	88.8634	21.4762	[-17.3795; 55.5786]	n/a	[2.9204; 39.5981]	34108.0731	527.8	9001
treeLikelihood.ASF	-348.4671	0.5005	5.9135	34.9691	-347.9381	[-369.1717; -332.5306]	n/a	[-357.7008; -336.0696]	1.29E+05	139.6	9001
treeLikelihood.COI	-576.5385	0.0947	2.9025	8.4244	-576.3543	[-591.5371; -567.0102]	n/a	[-582.5733; -571.3437]	19177.1382	938.7	9001
treeLikelihood.ENOLASE	-1771.192	0.9219	9.3033	86.551	-1772.576	[-1801.5195; -1747.0863]	n/a	[-1787.1689; -1754.7433]	1.77E+05	101.8	9001
treeLikelihood.HSP90	-1599.417	0.9851	8.7835	77.1496	-1595.888	[-1626.3787; -1583.0736]	n/a	[-1613.5203; -1587.1059]	2.26E+05	79.5	9001
treeLikelihood.NADHI	-1383.174	0.5157	6.2071	38.5275	-1383.666	[-1407.2072; -1362.4585]	n/a	[-1394.4965; -1370.5127]	1.24E+05	144.8	9001
TreeHeight.t:ASF	3.89E-03	6.25E-06	5.20E-04	2.71E-07	3.85E-03	[2.2499E-3; 6.7559E-3]	3.86E-03	[2.9578E-3; 4.9571E-3]	2595.2235	6936.6	9001
kappa.s:COX	16.671	0.2955	9.9872	99.7437	14.6351	[2.4976; 125.1793]	14.6255	[4.508; 34.1416]	15757.7349	1142.4	9001
kappa.s:NADH	7.6195	0.0751	2.9614	8.7699	7.0671	[2.0675; 32.9926]	7.1248	[2.931; 13.3633]	11566.6179	1556.4	9001
mutationRate.s:ASF	1.0571	6.67E-03	0.3644	0.1328	0.9988	[0.2404; 3.0826]	0.9986	[0.4194; 1.7718]	6027.3509	2986.7	9001
mutationRate.s:COI	1.3768	3.15E-03	0.2775	0.077	1.3517	[0.5849; 2.5658]	1.3493	[0.8631; 1.9294]	2318.4609	7764.6	9001
mutationRate.s:ENOLASE	1.2569	2.75E-03	0.1427	0.0204	1.2565	[0.7474; 1.8553]	1.2487	[0.9794; 1.5282]	6677.0694	2696.1	9001
mutationRate.s:HSP90	0.5051	2.98E-03	0.1024	0.0105	0.5009	[0.1713; 1.0887]	0.4945	[0.3125; 0.7083]	15191.2538	1185	9001
mutationRate.s:NADHI	1.131	2.12E-03	0.1552	0.0241	1.1279	[0.6033; 1.8197]	1.1202	[0.8283; 1.4328]	3351.4631	5371.4	9001
kappa1.s:ENOLASE	8.9322	0.0786	2.9984	8.9905	8.3708	[3.0714; 30.5644]	8.4829	[3.9207; 14.6789]	12386.0465	1453.4	9001
kappa1.s:HSP90	3.8662	0.0829	2.4213	5.8625	3.2785	[0.3945; 24.7326]	3.2505	[0.5734; 8.2262]	21103.0895	853.1	9001
kappa2.s:ENOLASE	2.3268	0.0248	0.9607	0.923	2.1529	[0.3781; 8.5736]	2.1469	[0.7453; 4.2003]	12020.6759	1497.6	9001
kappa2.s:HSP90	17.0593	0.3116	9.7615	95.2864	14.7929	[2.5399; 91.3563]	14.9013	[4.09; 36.0253]	18349.0987	981.1	9001
NeConstant.t:Australia	1.81E-03	8.73E-06	5.23E-04	2.73E-07	1.74E-03	[5.9562E-4; 4.5801E-3]	1.74E-03	[8.9843E-4; 2.8762E-3]	5013.9747	3590.4	9001
NeConstant.t:Canary	0.5207	0.023	0.9958	0.9915	0.2127	[2.1324E-3; 15.9]	0.2282	[2.1324E-3; 1.9875]	9628.9884	1869.6	9001
NeConstant.t:Europe	0.1118	4.27E-03	0.1978	0.0391	0.0532	[9.1283E-4; 3.9483]	0.055	[9.1283E-4; 0.3909]	8392.9793	2144.9	9001
NeConstant.t:NewZealand	1.0896	0.0341	1.6962	2.8771	0.5392	[1.5213E-3; 27.968]	0.513	[1.5213E-3; 3.8725]	7291.0314	2469.1	9001
NeConstant.t:NorthAmerica	1.5157	0.0362	2.0486	4.1969	0.8748	[0.0112; 53.3656]	0.8708	[0.0112; 5.0507]	5609.8104	3209	9001
NeConstant.t:SouthAfrica	3.84E-03	1.59E-05	9.45E-04	8.93E-07	3.70E-03	[1.5731E-3; 9.5885E-3]	3.73E-03	[2.261E-3; 5.818E-3]	5112.4863	3521.2	9001
*b_migrationConstant.t:NewZealand_to_Australia	7.1168	0.0803	2.8312	8.0159	6.8202	[0.8744; 21.8329]	6.557	[2.1601; 12.7592]	14494.3045	1242	9001
b_migrationConstant.t:Europe_to_Australia	4.7588	0.067	2.2262	4.9561	4.4587	[0.338; 13.6427]	4.2398	[0.8606; 9.0996]	16330.854	1102.3	9001
b_migrationConstant.t:Australia_to_SouthAfrica	4.3859	0.0575	2.0878	4.3589	4.0647	[0.163; 16.882]	3.8831	[0.8059; 8.5642]	13644.5694	1319.4	9001
b_migrationConstant.t:Europe_to_SouthAfrica	3.1981	0.0753	1.9776	3.911	2.8019	[0.0211; 14.7072]	2.5989	[0.0865; 6.8777]	26071.119	690.5	9001
b_migrationConstant.t:NorthAmerica_to_Australia	2.8604	0.0532	1.785	3.1864	2.5122	[0.0162; 11.3561]	2.3077	[0.1496; 6.6253]	16012.1468	1124.3	9001
b_migrationConstant.t:Canary_to_Australia	2.4973	0.0591	1.7756	3.1528	2.0955	[0.0266; 13.7787]	1.9026	[0.1011; 5.8995]	19973.6491	901.3	9001
b_migrationConstant.t:SouthAfrica_to_Australia	1.1988	0.0593	1.2381	1.5329	0.8104	[5.116E-5; 10.7166]	0.6654	[5.116E-5; 3.5073]	41246.2961	436.5	9001
b_migrationConstant.t:NewZealand_to_Europe	1.1715	0.0561	1.2118	1.4686	0.7986	[1.3213E-3; 15.9261]	0.6592	[1.3213E-3; 3.4833]	38594.3642	466.4	9001
b_migrationConstant.t:Europe_to_Canary	1.0611	0.0437	1.0386	1.0787	0.7708	[4.9054E-5; 7.8189]	0.5826	[1.6519E-4; 3.1883]	31868.7289	564.9	9001
b_migrationConstant.t:NewZealand_to_SouthAfrica	1.0559	0.0481	1.111	1.2343	0.6792	[5.8915E-4; 10.0676]	0.5565	[5.8915E-4; 3.3774]	33774.1199	533	9001
b_migrationConstant.t:Europe_to_NewZealand	1.0558	0.0445	1.0984	1.2066	0.7185	[1.1922E-4; 9.4185]	0.5839	[1.1922E-4; 3.1192]	29556.2322	609.1	9001
b_migrationConstant.t:Canary_to_Europe	1.0557	0.0368	1.0011	1.0021	0.7507	[2.5293E-4; 6.8595]	0.6042	[4.4652E-4; 3.1916]	24358.7387	739	9001
b_migrationConstant.t:Canary_to_NewZealand	1.0451	0.0374	1.0222	1.0449	0.7475	[4.7457E-4; 9.0483]	0.6028	[5.5138E-4; 2.998]	24053.8232	748.4	9001
b_migrationConstant.t:NewZealand_to_Canary	1.0435	0.0397	1.0162	1.0327	0.7264	[1.8202E-3; 8.8855]	0.5978	[3.4386E-3; 3.0566]	27484.9554	655	9001
b_migrationConstant.t:NorthAmerica_to_Europe	1.0368	0.0368	1.0026	1.0052	0.7423	[1.2054E-3; 8.2179]	0.6069	[1.5133E-3; 2.9721]	24284.9316	741.3	9001
b_migrationConstant.t:SouthAfrica_to_Europe	1.0286	0.0381	0.9789	0.9582	0.7431	[9.607E-6; 9.5051]	0.5921	[9.607E-6; 2.9713]	27302.4226	659.4	9001
b_migrationConstant.t:NorthAmerica_to_NewZealand	1.0275	0.0388	1.0082	1.0165	0.7137	[3.5944E-4; 7.0604]	0.5778	[3.5944E-4; 3.0617]	26652.5554	675.4	9001
b_migrationConstant.t:NewZealand_to_NorthAmerica	1.0238	0.0373	1.0275	1.0558	0.7075	[5.9062E-4; 7.9858]	0.5836	[5.9062E-4; 3.0357]	23734.3681	758.5	9001
b_migrationConstant.t:Australia_to_Canary	1.0159	0.0366	0.976	0.9525	0.7163	[7.2784E-4; 7.6225]	0.5864	[7.2784E-4; 3.0853]	25306.7173	711.4	9001
b_migrationConstant.t:Australia_to_Europe	1.0141	0.0505	1.0534	1.1096	0.6673	[5.3308E-5; 7.7427]	0.5358	[6.7193E-5; 3.0928]	41322.5061	435.6	9001
b_migrationConstant.t:NorthAmerica_to_SouthAfrica	1.0063	0.0365	1.0184	1.0372	0.6996	[1.4665E-3; 7.7548]	0.5573	[1.5509E-3; 2.9897]	23169.8228	777	9001
b_migrationConstant.t:Canary_to_SouthAfrica	0.9954	0.0356	0.9744	0.9495	0.7	[1.9182E-4; 6.4083]	0.5505	[1.9182E-4; 2.9513]	24038.6675	748.9	9001
b_migrationConstant.t:NorthAmerica_to_Canary	0.9953	0.0387	1.0066	1.0133	0.6779	[1.8847E-3; 8.4098]	0.5494	[1.8847E-3; 3.0312]	26564.3331	677.7	9001
b_migrationConstant.t:Australia_to_NewZealand	0.9914	0.0326	0.9781	0.9566	0.7268	[1.0392E-3; 7.8908]	0.5864	[1.0392E-3; 2.7681]	20008.4981	899.7	9001
b_migrationConstant.t:Australia_to_NorthAmerica	0.9802	0.0467	0.9668	0.9347	0.6874	[4.0638E-4; 7.4496]	0.5634	[8.2404E-4; 2.9071]	42062.8755	428	9001
b_migrationConstant.t:Canary_to_NorthAmerica	0.9774	0.0376	0.979	0.9583	0.6848	[1.4064E-3; 9.6053]	0.5559	[1.4064E-3; 2.9797]	26620.6777	676.2	9001
b_migrationConstant.t:Europe_to_NorthAmerica	0.973	0.0354	0.9529	0.9079	0.6995	[5.5813E-4; 8.7981]	0.5529	[1.3984E-3; 2.8995]	24802.7458	725.8	9001
b_migrationConstant.t:SouthAfrica_to_Canary	0.9655	0.0339	0.9628	0.927	0.6521	[7.4174E-4; 7.0515]	0.5429	[1.0325E-3; 2.9746]	22299.0508	807.3	9001
b_migrationConstant.t:SouthAfrica_to_NorthAmerica	0.9573	0.0406	0.9827	0.9657	0.6516	[1.817E-4; 9.575]	0.5108	[8.9674E-4; 2.9135]	30674.6091	586.9	9001
b_migrationConstant.t:SouthAfrica_to_NewZealand	0.9416	0.0369	0.9604	0.9224	0.6337	[5.9553E-4; 6.839]	0.5247	[8.3547E-4; 2.8346]	26555.3608	677.9	9001
Mascot	124.5497	0.3022	8.7221	76.0759	124.6123	[91.1853; 154.3462]	124.2415	[108.0367; 141.9192]	21609.1267	833.1	9001

*b_migration - migration rates are backwards in time, e.g. "b_migrationConstant.t:ASF_CC_mul.NewZealand_to_Australia" shows migration from Australia to New Zealand.
